# Regulatory Mechanisms of XBP1 in Tumorigenesis and Cancer Progression: Challenges and Therapeutic Strategies

**DOI:** 10.3390/ph19070993

**Published:** 2026-06-26

**Authors:** Haiyan Jiang, Zhanzhan Li, Jie Wang, Hualin Sun, Lei Qi

**Affiliations:** 1 Department of Emergency Medicine, Affiliated Hospital of Nantong University, Nantong University, Nantong 226001, China; jhy@ntu.edu.cn; 2 Jiangsu Key Laboratory of Tissue Engineering and Neuroregeneration, Key Laboratory of Neuroregeneration of Ministry of Education, Co-Innovation Center of Neuroregeneration, Medical School of Nantong University, Nantong University, Nantong 226001, China; 2524310026@stmail.ntu.edu.cn (Z.L.); 2022310019@stmail.ntu.edu.cn (J.W.)

**Keywords:** XBP1, endoplasmic reticulum stress, tumor microenvironment, immunosuppression, therapeutic resistance, targeted therapy

## Abstract

Endoplasmic reticulum (ER) stress is a common state of cellular adversity experienced by tumor cells under unfavorable conditions such as hypoxia, nutrient deprivation, and oncogene activation. As the most conserved signaling branch of the unfolded protein response (UPR), the inositol-requiring enzyme 1α (IRE1α)- X-box-binding protein 1 (XBP1) pathway plays a central role in sustaining tumor cell survival, driving malignant progression, and remodeling the tumor microenvironment (TME). XBP1, the terminal transcription factor of this pathway, finely orchestrates tumor cell fate through both its canonical and non-canonical functions. This review systematically summarizes the dual mechanisms of XBP1 in cancer: within cancer cells, XBP1 promotes proliferation, metastasis, and chemoresistance via metabolic reprogramming, anti-apoptotic proteins, and DNA repair; within immune cells (macrophages, dendritic cells, T cells), XBP1 fosters an immunosuppressive microenvironment, while also modulating cancer-associated fibroblasts, endothelial cells, and osteoclasts. Despite its therapeutic promise, several major unresolved questions remain, including the precise molecular switch governing XBP1’s pro-tumorigenic versus anti-tumorigenic functions, the functional divergence between XBP1u and XBP1s isoforms in different cellular contexts, and the lack of reliable predictive biomarkers for patient stratification. Key translational challenges involve the on-target toxicity of systemic XBP1/IRE1α inhibition due to its essential roles in normal tissues, the cell-type-specific and context-dependent effects that complicate therapeutic outcomes, and the limited selectivity and off-target effects of current inhibitors, as well as compensatory activation of other UPR branches that may drive adaptive resistance. Finally, this review discusses XBP1-targeted therapeutic strategies, including small-molecule inhibitors, nucleic acid-based drugs, immunotherapeutic combination approaches, and XBP1-based tumor vaccines, and provides perspectives on future research directions, aiming to establish a theoretical foundation for the development of more effective and precise XBP1-targeted therapies for tumorigenesis and cancer progression.

## 1. Introduction

Tumorigenesis and cancer progression are complex, multi-step processes driven by genetic and epigenetic alterations. During this process, tumor cells must activate a series of critical adaptive signalling pathways to survive the harsh tumor microenvironment (TME), characterised by hypoxia, nutrient deprivation, acidic conditions and oxidative stress. The endoplasmic reticulum (ER), a key organelle responsible for the synthesis, folding, assembly and secretion of secretory and membrane proteins, is essential for cellular homeostasis [1]. When the accumulation of unfolded or misfolded proteins in the ER exceeds its processing capacity, ER stress is induced. To cope with this stress and restore ER homeostasis, cells initiate a highly conserved adaptive response known as the unfolded protein response (UPR) [2]. Therefore, a detailed understanding of the regulatory mechanisms of the UPR is crucial for elucidating how tumor cells survive and progress under adverse conditions.

The UPR is primarily mediated by three ER transmembrane sensors: protein kinase R-like ER kinase (PERK), activating transcription factor 6 (ATF6), and inositol-requiring enzyme 1α (IRE1α). Among these, the IRE1α-X-box binding protein 1 (XBP1) axis represents the evolutionarily most conserved branch of the UPR [3]. Under homeostatic conditions, IRE1α remains inactive. However, upon ER stress, IRE1α dimerises and autophosphorylates, activating its cytoplasmic endoribonuclease domain, which subsequently cleaves the mRNA encoding *XBP1* at two specific sites, excising a 26-nucleotide intron. This unconventional splicing event causes a translational frameshift, generating a potent transcriptionally active spliced isoform, XBP1s (spliced XBP1) [4]. As a key transcription factor, XBP1s upregulates the expression of a broad range of genes involved in protein folding, secretion, ER biogenesis and lipid metabolism, thereby facilitating the restoration of ER homeostasis and promoting cell survival. Thus, the IRE1α-XBP1 pathway serves as a core adaptive mechanism enabling cells to survive ER stress.

While all three UPR branches—IRE1α-XBP1, PERK-eIF2α-ATF4, and ATF6—coordinately respond to ER stress, they exhibit distinct mechanistic features and functional outcomes. PERK-eIF2α-ATF4 signaling primarily attenuates global protein translation to reduce ER protein-folding load, while also regulating redox homeostasis, amino acid metabolism, and apoptosis through its downstream effector CHOP [1]. ATF6, upon ER stress, translocates to the Golgi where it is proteolytically activated and upregulates genes encoding ER chaperones and ERAD components to enhance folding capacity [1]. In contrast, the IRE1α-XBP1 pathway is unique in its dual enzymatic activities and its unconventional *XBP1* mRNA splicing mechanism, which generates the potent transcription factor XBP1s. First, XBP1s regulates the broadest repertoire of UPR target genes, encompassing protein folding, secretion, ER biogenesis, lipid metabolism, and redox homeostasis, making it a central hub in the adaptive stress response [5]. Second, unlike the stress-restricted activation of PERK and ATF6, XBP1 is aberrantly and persistently activated in multiple cancer types through oncogenic drivers such as c-Myc and HIF1α, establishing a dependency that cancer cells exploit for survival and progression [6,7,8]. Third, XBP1 exerts profound cell-non-autonomous functions by remodeling the tumor microenvironment, including reprogramming tumor-associated macrophages, inducing T cell exhaustion, and promoting myeloid-derived suppressor cell activation [9]. Fourth, the druggable nature of IRE1α’s kinase and RNase domains offer a tangible opportunity for pharmacological intervention [10]. Collectively, these features establish XBP1 as a central signaling node and an attractive target for cancer therapy.

Epidemiological evidence from large-scale public cohorts has increasingly linked aberrant XBP1 expression to patient outcomes across multiple cancer types, underscoring its clinical relevance as a prognostic biomarker. Integrative analyses of The Cancer Genome Atlas (TCGA) datasets have revealed that XBP1 exhibits distinct prognostic associations depending on tumor context. In ovarian cancer, meta-analysis of TCGA and HAS cohorts identified low *XBP1* mRNA expression as a predictor of poor overall survival [11]. Another study demonstrated that high XBP1 expression significantly benefits both overall survival and disease-free survival in ovarian cancer patients, with XBP1 genomic alterations closely correlated with antitumor immunity [12]. In non-small cell lung cancer, the spliced XBP1s isoform in TCGA datasets demonstrated that XBP1s expression predicts poor survival [13]. In gastric cancer, a protein-based prognostic model incorporating XBP1 was established using TCGA and TCPA data, showing that high-risk scores are associated with worse survival [14]. In colon cancer, an endoplasmic reticulum stress-responsive gene prognostic model that includes XBP1 effectively predicts patient outcomes and immunotherapy response [15]. In endometrial cancer, an ER stress-based risk signature comprising TRIB3, CREB3L3, XBP1, and PPP1R15A was constructed from TCGA data, demonstrating reliable predictive capability for patient prognosis and immune correlation [16]. In esophageal squamous cell carcinoma, single-cell and bulk RNA-seq analyses from TCGA and paired patient samples revealed that overactivation of XBP1 in plasma cells correlates with higher tumor grade and worse survival, while also linking XBP1 to innate immune signaling [17]. Collectively, these large-scale transcriptomic and prognostic analyses establish XBP1 as a context-dependent prognostic indicator across diverse malignancies, highlighting its potential utility both as a biomarker for patient stratification and as a rational therapeutic target, though its functional and prognostic significance must be interpreted within specific tumor types and molecular subtypes.

In cancers, the UPR, and particularly the IRE1α-XBP1 pathway, is frequently aberrantly and persistently activated due to sustained oncogene activation (e.g., c-Myc) [18], hypoxic microenvironments [8], and chemotherapy-induced stress [19]. Initially, XBP1 was viewed as a ‘bodyguard’ that promotes tumor cell survival, helping cancer cells withstand hostile environments. Nevertheless, accumulating evidence in recent years has revealed that the role of XBP1 in cancer is far more complex and multifaceted. It not only directly drives malignant phenotypes, including proliferation, invasion, metastasis and drug resistance, via cell-intrinsic mechanisms, but also profoundly influences tumor progression and therapeutic responses through cell-non-autonomous mechanisms by modulating various immune and stromal cells within the tumor microenvironment [18,19,20,21]. For instance, XBP1 activation not only remodels the metabolism of tumor cells themselves but also fosters an immunosuppressive microenvironment by promoting M2 polarisation of tumor-associated macrophages and recruitment of myeloid-derived suppressor cells, thereby enabling tumors to evade immune surveillance [18,20]. Furthermore, aberrant XBP1 activation in T cells and dendritic cells impairs their antitumor immune functions, further accelerating tumor progression [21]. Collectively, these findings establish XBP1 as a critical molecular hub linking tumor cell-intrinsic properties with the external microenvironment, playing a multidimensional role in malignant tumor progression.

Consequently, XBP1 has evolved from a simple stress-adaptive molecule into a key nexus connecting tumor cell-intrinsic features with the external milieu. Given its multifaceted and central roles in tumorigenesis and progression, XBP1 and its upstream regulator IRE1α have emerged as highly promising anticancer therapeutic targets. To date, various small-molecule inhibitors and gene therapy strategies targeting the IRE1α-XBP1 pathway have been developed, demonstrating encouraging antitumor efficacy in preclinical studies [18,19,20]. However, translating these fundamental research findings into effective clinical therapies faces numerous challenges, including how to precisely modulate this pathway to balance its pro-survival and pro-death functions, how to overcome the functional heterogeneity of XBP1 in different cell types, and how to develop inhibitors with high specificity and low toxicity. Therefore, a thorough analysis of these challenges is critical for the clinical translation of XBP1-targeted therapies.

This review aims to comprehensively dissect the complex regulatory network of XBP1 in cancer, systematically delineate its multifaceted functions in both tumor cells and the tumor microenvironment, discuss the major challenges currently associated with XBP1-targeted therapies, and provide a perspective on future XBP1-based therapeutic strategies. By integrating relevant research advances, this article seeks to offer new insights into the biological functions of XBP1 and its potential as a cancer therapeutic target. Through a systematic synthesis of current research progress, this review aspires to provide a robust theoretical foundation for the optimisation and innovation of XBP1-based cancer therapeutic strategies.

## 2. Biological Basis of XBP1

As a key transcription factor of the UPR, XBP1 is activated under precise control by endoplasmic reticulum stress signals and plays a central role in maintaining cellular homeostasis and determining cell fate. A deep understanding of its regulatory mechanisms is of great significance for elucidating the pathogenesis of related diseases.

### 2.1. Endoplasmic Reticulum Stress and the Unfolded Protein Response

The ER is a crucial organelle in eukaryotic cells responsible for the synthesis, folding, and transport of secreted and membrane proteins [1]. Its functional homeostasis is essential for cell survival. Endoplasmic reticulum stress (ER stress) occurs when the protein-folding capacity of the ER cannot meet the synthetic demand [22]. Various pathophysiological conditions, such as hypoxia, nutrient deprivation, Ca^2+^ homeostasis imbalance, oxidative stress, excessive protein synthesis rates, or the accumulation of mutant proteins, can disrupt ER homeostasis and thus induce ER stress [2,4,23]. To cope, cells activate the UPR, which is primarily mediated by three ER transmembrane sensors: IRE1α, PERK, and ATF6α. Among these, the IRE1α–XBP1 axis is the evolutionarily most conserved branch and has emerged as a central node linking ER stress to transcriptional reprogramming, metabolic adaptation, and immune modulation in cancer.

Under homeostatic conditions, IRE1α remains inactive, bound by the chaperone BiP/GRP78. Upon ER stress, BiP dissociates, allowing IRE1α to dimerize, autophosphorylate, and activate its cytoplasmic endoribonuclease domain, which then catalyzes the unconventional splicing of *XBP1* mRNA to generate the active transcription factor XBP1s [4]. XBP1s upregulates genes involved in protein folding, ERAD, lipid metabolism, and ER biogenesis, thereby restoring proteostasis and promoting cell survival. However, the UPR is not unidirectionally pro-survival. The transition from adaptive UPR to apoptosis is governed by multiple pathways that integrate stress intensity and duration. First, the PERK–eIF2α–ATF4–CHOP axis represents a major pro-apoptotic arm. PERK-mediated eIF2α phosphorylation paradoxically enables selective translation of ATF4, which transcriptionally upregulates CHOP. CHOP promotes apoptosis by suppressing anti-apoptotic BCL-2 family members, upregulating pro-apoptotic BIM and PUMA, and driving ROS accumulation through enhanced protein synthesis [24]. Second, IRE1α can initiate apoptosis through two distinct mechanisms: (i) it recruits TRAF2 to activate ASK1–JNK signaling, which phosphorylates and inactivates BCL-2 while activating BIM; and (ii) its RNase domain, when hyperactivated, promiscuously degrades ER-localized mRNAs and microRNAs in a process termed regulated IRE1α-dependent decay (RIDD). RIDD relieves repression of pro-apoptotic factors such as caspase-2 and TXNIP by cleaving miRNAs, thereby sensitizing cells to apoptotic stimuli [24,25]. Third, ATF6α primarily supports survival but can cooperate with CHOP under prolonged stress. Importantly, cancer cells frequently adapt to chronic ER stress by upregulating anti-apoptotic proteins via STAT3 and NFκB, which are themselves activated downstream of IRE1α and PERK, thereby tipping the balance away from apoptosis and toward malignancy [24]. Thus, the UPR operates as a dynamic “life-or-death switch,” the precise regulation of which is critical in determining cell fate under stress conditions.

### 2.2. Post-Transcriptional Regulation of XBP1

**Figure 1:**
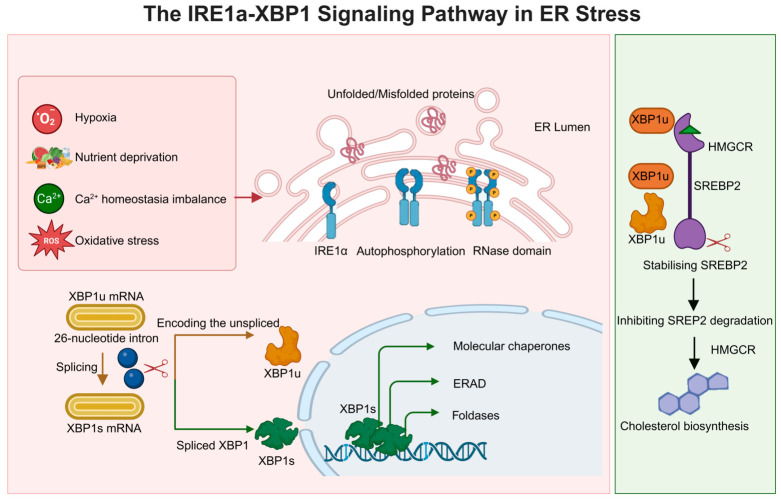
Under stress stimuli such as hypoxia, nutrient deprivation, Ca^2+^ imbalance, reactive oxygen species (ROS), and oxidative stress, endoplasmic reticulum (ER) stress is induced. Activated IRE1α endoribonuclease excises a 26-nucleotide intron from unspliced *Xbp1* mRNA (Xbp1u), generating spliced *Xbp1s* mRNA, which is translated into the active transcription factor XBP1s. XBP1s translocates to the nucleus and upregulates target genes involved in protein folding, ER-associated degradation (ERAD), and lipid metabolism, thereby restoring ER homeostasis. Additionally, the unspliced isoform XBP1u stabilizes SREBP2 by inhibiting its ubiquitin-mediated degradation, which in turn enhances cholesterol biosynthesis (e.g., via HMGCR). Thus, the IRE1α–XBP1 axis integrates metabolic regulation with proteostasis maintenance. Created in BioRender. Sun, H. (2026). https://BioRender.com/r4kv1j5.

Among the three signaling branches of the UPR, the non-canonical splicing pathway of XBP1, mediated by IRE1α, represents the most conserved branch and serves as a central link between ER stress and transcriptional reprogramming. The *XBP1* gene is first transcribed into full-length mRNA encoding the unspliced XBP1 protein (XBP1u). For a long time, XBP1u was considered merely a precursor to XBP1-s, and its functions were severely underestimated; however, recent studies have demonstrated that XBP1u itself possesses unique biological functions and participates in various processes, including autophagy and tumorigenesis [26]. Upon ER stress, the transmembrane protein IRE1α, acting as a key sensor, is activated through autophosphorylation of its kinase domain, which in turn activates its *C-* ribonuclease (RNase) domain [27]. The activated IRE1α recognizes specific stem-loop structures within *XBP1* mRNA and precisely excises a 26-nucleotide intron [4]. This unconventional splicing event causes a frameshift in the open reading frame of *XBP1* mRNA, leading to the translation of a full-length protein with a more stable structure and significantly enhanced transcriptional activation capacity: spliced XBP1 (XBP1s). XBP1s and XBP1u differ in their *C-* amino acid sequences, and it is this difference that endows XBP1s with potent transcriptional regulatory functions, enabling it to activate the expression of a range of downstream UPR target genes, thereby promoting cell survival, enhancing protein-folding capacity, and regulating metabolic reprogramming under stress conditions [4] (Figure 1). Therefore, the precise conversion from XBP1u to XBP1s represents a critical molecular switch through which cells transition from sensing ER stress to initiating an adaptive transcriptional response, and its dysregulation is closely associated with various diseases, particularly the onset and progression of malignant tumors (Table 1). Consequently, targeting this finely tuned splicing regulatory mechanism offers a highly promising new strategy for intervening in the progression of related diseases.

### 2.3. Alternative Regulatory Mechanisms of XBP1 Beyond IRE1α-Mediated Splicing

Beyond the canonical IRE1α-mediated splicing, XBP1 expression and activity are subject to multilayer regulation by microRNAs (miRNAs), RNA-binding proteins (RBPs), and epigenetic modifications, which collectively fine-tune its functions in cancer. At the post-transcriptional level, multiple miRNAs directly target *XBP1* mRNA to modulate its expression. In diffuse large B-cell lymphoma, miR-320a binds to the 3′UTR of XBP1, and its suppression by the long non-coding RNA LINC00963 relieves this inhibition, leading to XBP1 upregulation and subsequent ER stress-mediated apoptosis and autophagy [28]. RNA-binding proteins also critically modulate *XBP1* expression and splicing. The RNA ligase RtcB catalyzes the final ligation step of *XBP1* mRNA splicing following IRE1α-mediated intron excision; RtcB is essential for generating mature XBP1s and has been implicated in cancer cell survival and protein homeostasis [29]. At the epigenetic level, XBP1 expression is regulated by chromatin modifiers. ALKBH5-mediated N^6^-methyladenosine (m^6^A) modification of *XBP1* mRNA facilitates its expression and promotes non-small cell lung cancer progression through the IL-6/JAK/STAT3 pathway [30]. These findings collectively demonstrate that XBP1 is governed by a complex regulatory network beyond IRE1α-mediated splicing, encompassing miRNA-mediated silencing, RBP-dependent mRNA processing, and epigenetic modulation.

## 3. Intrinsic Mechanisms of XBP1 Action in Tumor Cells

**Figure 2:**
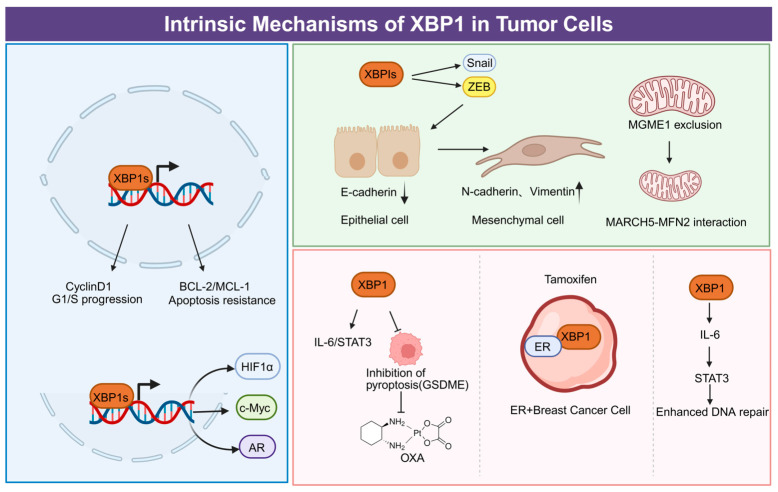
XBP1 promotes tumor cell proliferation, survival, metastasis, and therapy resistance through cell-intrinsic mechanisms. XBP1s upregulates Cyclin D1 to accelerate cell cycle progression and anti-apoptotic proteins (BCL-2, MCL-1) to block apoptosis. It synergizes with HIF1α, c-Myc, and androgen receptor (AR) signaling. XBP1 induces epithelial–mesenchymal transition (EMT) by upregulating Snail and ZEB transcription factors, while XBP1u contributes to metabolic reprogramming (aerobic glycolysis/Warburg effect) and mitochondrial fission (via the MARCH5–MFN2 axis). XBP1 confers resistance to chemotherapy (cisplatin, oxaliplatin), endocrine therapy (tamoxifen), and radiotherapy by activating the IL-6/STAT3 pathway, enhancing DNA repair, and inhibiting GSDME-mediated pyroptosis. Created in BioRender. Sun, H. (2026). https://BioRender.com/r4kv1j5.

As a key transcription factor within the UPR signalling pathway, XBP1 orchestrates a complex, multi-dimensional regulatory network that underpins its intrinsic functions in tumor cells. Functionally, XBP1 not only promotes tumor cell proliferation and survival by directly regulating the expression of cell cycle-related genes and anti-apoptotic factors, but also induces epithelial–mesenchymal transition (EMT), thereby enhancing tumor cell invasion and metastatic capacity. Furthermore, aberrant activation of XBP1 is extensively involved in the development of resistance to chemotherapy, radiotherapy and endocrine therapy. A deep understanding of these intrinsic mechanisms is therefore of great significance for elucidating its pro-tumorigenic functions and for developing targeted therapeutic strategies (Figure 2).

### 3.1. Promotion of Tumor Cell Proliferation and Survival

In multiple solid tumors, overexpression of XBP1, particularly its active spliced isoform XBP1s, has emerged as a key molecular hallmark associated with increased malignancy and poor patient prognosis [31]. The underlying mechanisms by which XBP1 promotes tumor growth are remarkably complex and diverse, primarily encompassing universally conserved pathways, such as direct regulation of the cell cycle and inhibition of apoptosis, and cancer-type-specific adaptations that reflect the unique metabolic and signaling dependencies of individual malignancies. Consequently, aberrant activation of XBP1 constitutes a core molecular basis for the survival advantage of tumor cells.

#### 3.1.1. Direct Regulation of Cell Cycle and Anti-Apoptotic Genes

As a conserved transcriptional function across multiple cancer types, XBP1s directly binds to and activates the promoters of key cell cycle regulators, such as XBP1s directly binding to the Cyclin D1 promoter to activate its transcription, thereby accelerating the G1-to-S phase transition—a mechanism validated in both colorectal cancer [31] and hepatocellular carcinoma [32]. Concurrently, XBP1s upregulates the expression of anti-apoptotic protein family members, including MCL-1 and BCL-2 through multiple downstream effectors [33,34], effectively enhancing the resistance of tumor cells to both intrinsic and extrinsic apoptotic signals, thus ensuring their survival within a stressful microenvironment. This anti-apoptotic function appears to be broadly conserved. Moreover, XBP1 modulates tumor progression through a negative feedback loop: it interacts with the E3 ubiquitin ligase Fbw7, and overexpression of XBP1s downregulates Fbw7 expression, whereas Fbw7 can mediate XBP1s degradation—a mechanism that potentially promotes tumor progression in various cancers [35]. Collectively, these findings indicate that XBP1s establishes a dual safeguard for promoting tumor proliferation by co-regulating cell cycle progression and apoptotic resistance, representing a core, cancer-independent oncogenic program.

#### 3.1.2. Synergy with Oncogenic Signalling Pathways and Enhanced Stress Adaptation

The synergistic interactions between XBP1s and specific oncogenic pathways exhibit considerable cancer-type specificity, reflecting the distinct genetic drivers of different malignancies. XBP1s does not act in isolation; instead, it synergises with multiple key oncogenic signalling pathways to collectively shape the malignant phenotype of tumor cells. In triple-negative breast cancer (TNBC), XBP1s forms a transcriptional complex with hypoxia-inducible factor 1α (HIF1α), co-activating downstream target genes such as VEGFA, thereby enhancing angiogenesis and survival adaptability under hypoxic conditions [8]. Furthermore, XBP1s cooperates with c-Myc to regulate the expression of glycolysis-related genes, promoting metabolic reprogramming to meet the energy and biosynthetic demands of rapid proliferation [18]. Of note, recent studies have revealed crosstalk between XBP1 and androgen receptor (AR) signalling: in prostate cancer, AR signalling induces XBP1 expression, establishing a positive feedback loop that co-regulates a range of genes involved in proliferation and stress adaptation [36]. In pancreatic cancer, activated XBP1s directly binds to the promoters of genes such as *IL-6*, activating the JAK–STAT3 signalling pathway, which not only promotes tumor cell proliferation but also mediates cancer cachexia-associated muscle wasting [37]. The IL-6/STAT3 axis has also been implicated in hepatocellular carcinoma and non-small cell lung cancer [30,38]. While XBP1–STAT3 crosstalk is observed across multiple cancer types, its upstream triggers and downstream consequences exhibit tissue-specific features. Additionally, emerging evidence highlights XBP1 integration with KRAS, PI3K/AKT/mTOR, and Wnt/β-catenin signaling. In KRAS-mutant colorectal cancer, mutant KRAS drives ART1-mediated MARylation of GRP78, which sustains IRE1α–XBP1 activation to maintain ER homeostasis and support tumor cell survival, making KRAS-mutant cells particularly dependent on this axis [39]. Regarding PI3K/AKT/mTOR signaling, studies in melanoma have shown that activation of the IRE1α–XBP1 pathway intersects with PI3K/AKT/mTOR to regulate autophagy and apoptosis, while in hormone receptor-positive breast cancer, the IRE1–XBP1 axis contributes to endocrine resistance through crosstalk with PI3K/AKT/mTOR signaling [40,41]. The XBP1–Wnt/β-catenin interaction exhibits remarkable context dependency: in hematopoietic stem and progenitor cells, IRE1α–XBP1 signaling represses Wnt–β-catenin pro-leukemogenic programs, thereby safeguarding against acute myeloid leukemia [42]; conversely, in hepatocellular carcinoma, XBP1s transactivates LEF1 (a key β-catenin co-factor) by binding to its promoter and physically interacts with LEF1 to enhance classical Wnt signaling, rendering HCC with hyperactivated Wnt/LEF1 selectively vulnerable to IRE1α inhibition [43]. This functional duality underscores that the outcome of XBP1–Wnt crosstalk is highly tissue- and context-dependent. Thus, by forming a regulatory network with multiple core oncogenic pathways, XBP1s profoundly enhances the ability of tumor cells to cope with a hostile microenvironment.

#### 3.1.3. Regulation of Metabolic Reprogramming

Metabolic reprogramming is a central hallmark of tumor cells, and XBP1 plays an indispensable role in this process. While XBP1-driven metabolic regulation is broadly observed across cancers, the specific pathways and isoform contributions are often cancer-type-specific. The two isoforms of XBP1—XBP1u (unspliced) and XBP1s (spliced)—participate in metabolic regulation through distinct mechanisms. In hepatocellular carcinoma, XBP1u interacts with SREBP2, inhibiting its ubiquitin-mediated degradation and thereby stabilising SREBP2, which upregulates the expression of HMGCR—the rate-limiting enzyme in cholesterol biosynthesis—ultimately promoting tumor cell proliferation [44]. This XBP1u-dependent cholesterol biosynthesis pathway appears to be particularly relevant in liver cancer, where lipid metabolism is a key driver of tumorigenesis. This finding reveals an important function of XBP1u in tumor metabolism that is independent of XBP1s. In contrast, XBP1s promotes aerobic glycolysis (the Warburg effect) and lactate production in non-small cell lung cancer by upregulating PDK1 expression, thereby inducing EMT and enhancing tumor cell invasion and metastasis [45]. Additionally, XBP1s regulates fatty acid metabolism; for example, in colon cancer, XBP1s upregulates LOXL2, which in turn activates the IRE1α–XBP1 axis, promoting EMT [46]. Moreover, XBP1 influences metabolic adaptation by modulating mitochondrial dynamics and autophagy. Moreover, XBP1 influences metabolic adaptation by modulating mitochondrial dynamics and autophagy in a context-dependent manner. In melanoma, XBP1 promotes MARCH5-mediated degradation of MFN2, inducing mitochondrial fission and mitophagy, thereby maintaining mitochondrial function and resisting ER stress-induced cell death [47]. In hepatocytes, XBP1 deficiency impairs mitophagy, leading to mitochondrial DNA release, activation of the cGAS–STING pathway, and subsequent pyroptosis and inflammation [3]—underscoring the critical role of XBP1 in maintaining mitochondrial homeostasis. In diffuse large B-cell lymphoma, XBP1 expression is closely correlated with autophagic flux, influencing cell survival under stress conditions [28]. Collectively, these results demonstrate that XBP1 provides the necessary material and energy foundations for rapid tumor cell proliferation through fine-tuned regulation of metabolic reprogramming and organelle homeostasis, but the specific metabolic axes engaged are largely dictated by the tissue of origin and the genetic makeup of the tumor.

In summary, XBP1 establishes a powerful pro-survival and pro-proliferative programme within tumor cells through a multi-tiered regulatory network that includes direct control of the cell cycle and apoptosis, synergy with core oncogenic pathways, and precise modulation of cellular metabolism and organelle homeostasis. These mechanisms collectively reveal the central driving role of XBP1 in cancer development and progression, providing a robust theoretical basis for its consideration as a potential therapeutic target while cautioning that the efficacy of XBP1-targeted strategies may vary across cancer types due to these context-specific dependencies. Therefore, an in-depth dissection of the complexity of the XBP1 regulatory network will open new avenues for the development of novel anti-cancer strategies targeting this molecule.

### 3.2. Induction of Epithelial–Mesenchymal Transition (EMT) and Tumor Metastasis

As a key driver of tumor metastasis, XBP1 exerts its pro-metastatic effects through multiple sophisticated mechanisms that encompass both the remodelling of tumor cell properties and the regulation of the tumor microenvironment. These interconnected mechanisms collectively establish a network that facilitates tumor dissemination. Critically, converging in vivo evidence from diverse cancer models has established that XBP1 is not merely a regulator of metastatic traits in vitro but is genuinely required for successful metastatic colonization in vivo.

First, XBP1 directly regulates core transcription factors involved in EMT. In breast cancer cells, XBP1s directly upregulates Snail expression, which in turn suppresses the epithelial marker E-cadherin and induces the mesenchymal markers N-cadherin and Vimentin, a process critical for promoting breast cancer cell migration and invasion [48]. In hepatocellular carcinoma, in vivo experiments have confirmed that XBP1s overexpression promotes EMT and metastasis [49]. Notably, the involvement of XBP1 in EMT extends beyond this axis. For instance, palmitic acid activates the IRE1α–XBP1 signalling pathway to upregulate ZEB family transcription factors, thereby transcriptionally repressing desmoplakin expression and ultimately promoting the migration of liver and breast cancer cells [50]. Furthermore, in bladder cancer, circ-BIRC6 modulates XBP1 expression by sponging miR-495-3p, thereby influencing EMT-related proteins and accelerating tumor progression [51]. In non-small cell lung cancer, the IRE1α–XBP1 signalling axis promotes glycolysis and lactate production by upregulating pyruvate dehydrogenase kinase 1 (PDK1), which subsequently induces Snail expression and the EMT programme [45]. Collectively, these findings establish XBP1 as a central regulator of EMT across multiple cancer types through diverse upstream signals and downstream effectors.

Second, XBP1 enhances tumor invasiveness by activating multiple pro-metastatic signalling pathways and effector molecules. In oesophageal squamous cell carcinoma (ESCC), XBP1s upregulates matrix metalloproteinase-9 (MMP-9) expression, thereby increasing the degradation of the extracellular matrix and facilitating tumor invasion [45]. Similarly, in oral squamous cell carcinoma, XBP1 overexpression is closely associated with the upregulation of AXL, PI3K, MMP1, MMP3 and uPA, which collectively promote tumor invasion [52]. Moreover, in tongue squamous cell carcinoma, chemotherapy-induced tumor cell death creates an XBP1-driven microenvironment enriched in amphiregulin (AREG) and basic fibroblast growth factor (bFGF), which in turn activates NF-κB signalling to promote the proliferation and repopulation of surviving tumor cells [53]. In lung cancer, XBP1 promotes the invasion and metastasis of non-small cell lung cancer by upregulating insulin-like growth factor-binding protein 3 (IGFBP3) expression [54]. Gain- and loss-of-function experiments have further demonstrated that XBP1s protein overexpression promotes cell invasion, migration and metastasis both in vitro and in vivo in NSCLC models [54]. These observations indicate that XBP1 can directly or indirectly remodel the interplay between tumor cells and the extracellular matrix while also responding to chemotherapeutic stress, thereby driving malignant progression.

Third, XBP1 influences tumor cell motility through the regulation of lipid metabolism, mitochondrial function and membrane fluidity. In hepatocellular carcinoma cells, XBP1 modulates phospholipid metabolism, thereby affecting membrane fluidity and signal transduction to promote cell migration [55]. Interestingly, XBP1u also plays a distinct role in this context. In colorectal cancer, XBP1u inhibits the mitochondrial translocation of mitochondrial genome maintenance exonuclease 1 (MGME1), leading to reduced mitochondrial number and metabolic reprogramming. This metabolic adaptation indirectly promotes tumor cell proliferation and invasion [55]. Concurrently, XBP1 is involved in regulating mitochondrial function in response to endoplasmic reticulum stress. For example, in melanoma, XBP1 promotes the transcription of the E3 ubiquitin ligase MARCH5, which mediates the degradation of the mitochondrial fusion protein MFN2, thereby inducing mitochondrial fission and mitophagy. This process helps maintain mitochondrial function and confers resistance to endoplasmic reticulum stress in tumor cells [56]. Collectively, these studies reveal that XBP1 functions not only as a transcription factor but also as a key node in cellular metabolic reprogramming, coordinating lipid homeostasis and mitochondrial dynamics to provide a metabolic foundation for tumor cell migration and survival.

Finally, XBP1 actively participates in the regulation of tumor-associated signalling pathways, thereby establishing positive feedback loops that promote tumor progression. In colorectal cancer, XBP1s directly binds to and inhibits the transcriptional activity of the tumor suppressor TAp73, thereby promoting cell proliferation and colony formation [57]. Simultaneously, XBP1s enhances the malignant phenotypes of various tumors, including melanoma, hepatocellular carcinoma and non-small cell lung cancer, by activating the IL-6/STAT3 signalling pathway [30,38]. The IL-6/STAT3 axis is one of the core pathways mediating XBP1-driven tumor progression. In nasopharyngeal carcinoma, XBP1 expression is closely correlated with IL-6/STAT3 pathway activation, which promotes tumor lymphangiogenesis and metastasis [38]. Moreover, a circular RNA (circFAM13B) localized to the endoplasmic reticulum has been shown to restrain nasopharyngeal carcinoma lymphatic metastasis through downregulating XBP1, providing additional in vivo evidence for the requirement of XBP1 in metastatic spread [38]. Additionally, crosstalk exists between XBP1 and AR signalling. In prostate cancer, AR signalling induces XBP1 expression, whereas XBP1 in turn enhances AR transcriptional activity, forming a positive feedback regulatory network that promotes tumor cell proliferation [36]. A synthetic lethal relationship has also been identified between XBP1 and the MYC oncogene. MYC drives the activation of the IRE1α–XBP1 pathway, and targeting this pathway selectively inhibits MYC-driven breast cancer growth [58]. In summary, through crosstalk with multiple key oncogenic signalling pathways (e.g., TAp73, IL-6/STAT3, AR and MYC), XBP1 establishes a complex regulatory network that amplifies its pro-tumorigenic effects in a multidimensional manner.

In conclusion, XBP1 drives tumor metastasis through multidimensional and multilevel mechanisms, including direct regulation of EMT transcription factors, activation of pro-metastatic signalling pathways, remodelling of metabolic and mitochondrial functions, and integration of diverse oncogenic signalling networks. Crucially, in vivo evidence confirms that XBP1 is genuinely required for successful metastatic colonization, positioning it as a promising therapeutic target for anti-metastatic intervention. These findings highlight the central role of XBP1 in tumor metastasis and provide a robust theoretical foundation for the development of anti-metastatic therapeutic strategies targeting XBP1.

### 3.3. Mediation of Resistance to Chemotherapy, Radiotherapy, and Endocrine Therapy

Activation of XBP1 represents a key mechanism by which tumor cells mount an adaptive stress response when faced with exogenous therapeutic pressure, thereby giving rise to treatment resistance. This resistance mechanism is broadly observed across multiple therapeutic modalities, including chemotherapy, endocrine therapy, and radiotherapy.

#### 3.3.1. Chemotherapy Resistance

In the development of chemotherapy resistance, XBP1 confers survival advantages to tumor cells through various intricate mechanisms. For instance, in non-small cell lung cancer, a study has revealed that the splicing factor CPSF6 mediates shortening of the 3′ untranslated region (3′UTR) of *XBP1* mRNA, leading to increased transcript stability. This alteration subsequently suppresses cisplatin (DDP)-induced ER stress, thereby enhancing tumor cell resistance to cisplatin [19]. This mechanism underscores the critical role of RNA processing in regulating XBP1 expression and chemosensitivity. Thus, post-transcriptional regulation of XBP1 constitutes a pivotal upstream event in the establishment of chemotherapy resistance.

Furthermore, XBP1 can mediate chemotherapy resistance by inhibiting specific forms of cell death. Specifically, in colorectal cancer, a positive feedback loop composed of CircPDIA3/miR-449a/XBP1 has been identified. This loop effectively inhibits GSDME-mediated pyroptosis by suppressing the palmitoylation of the GSDME protein, thereby blocking oxaliplatin (OXA)-induced tumor cell death and ultimately leading to chemotherapy resistance [59]. This finding indicates that XBP1 not only regulates classical cell survival pathways but also directly intervenes in the execution phase of cell death. These discoveries link XBP1 to pyroptosis, a newly recognised form of programmed cell death, offering novel perspectives for overcoming chemotherapy resistance. Beyond pyroptosis, other non-apoptotic cell death pathways, such as ferroptosis—driven by lipid peroxidation and GPX4 dysregulation—are increasingly recognized as therapeutic targets in neurological disorders, suggesting that XBP1 might also influence ferroptosis sensitivity in cancer cells under ER stress [60].

#### 3.3.2. Endocrine Therapy Resistance

In oestrogen receptor-positive (ER^+^) breast cancer, XBP1 and the ER engage in a close and complex interplay, forming a positive feedback loop that drives resistance to endocrine therapy. On one hand, activated XBP1s enhances the transcriptional activity of ER; on the other hand, ER signalling itself induces XBP1 expression [61]. This synergistic interaction greatly promotes tumor cell tolerance to endocrine therapeutic agents such as tamoxifen. Specifically, XBP1 confers tamoxifen resistance by upregulating the expression of cell cycle-related genes, including *RRM2* and *CDC6* [62]. This loop reveals how ER^+^ breast cancer cells exploit the ER stress response to sustain their growth signals, thereby evading attack by endocrine therapies.

Intervention strategies targeting this pathway have shown potential for reversing drug resistance. Studies have demonstrated that miR-770-5p can directly target and inhibit XBP1 expression, thereby restoring the sensitivity of ER^+^ breast cancer cells to tamoxifen [63]. Moreover, the crosstalk between XBP1 and the ER signalling axis is subject to regulation by additional molecules. For example, the nuclear receptor coactivator NCOA3 is a direct transcriptional target of XBP1. The XBP1–NCOA3 axis forms a positive feedback loop that not only maintains high XBP1 expression but also activates the PERK–eIF2α–ATF4 signalling pathway, collectively promoting endocrine therapy resistance [64]. Therefore, targeting XBP1 or its downstream effectors may represent an effective strategy to overcome endocrine therapy resistance. In summary, the crosstalk between XBP1 and the ER signalling pathway, along with its downstream effector network, constitutes the core mechanism underlying endocrine therapy resistance in ER^+^ breast cancer.

#### 3.3.3. Radiotherapy Resistance

Activation of XBP1 also plays a critical role in radiotherapy resistance in tumors. At the level of DNA damage response, emerging evidence indicates that XBP1 directly participates in the regulation of DNA repair machinery. In colorectal cancer, the ER-resident protein PRKCSH is upregulated following ionizing radiation and promotes radioresistance by activating the IRE1α/XBP1s signaling axis, which in turn reduces p53 ubiquitination and degradation, thereby enhancing DNA repair capacity and conferring resistance to radiotherapy [65]. In human papillomavirus-negative (HPV^−^) oropharyngeal carcinoma, radiotherapy-induced endoplasmic reticulum stress activates XBP1, which in turn promotes the secretion of the key inflammatory cytokine interleukin-6 (IL-6). The secreted IL-6 activates the STAT3 signaling pathway in an autocrine or paracrine manner, thereby enhancing the DNA double-strand break repair capacity of tumor cells and ultimately leading to radiotherapy resistance [66]. Furthermore, in oropharyngeal carcinoma, activation of the epidermal growth factor receptor (EGFR) potentiates both the PERK–eIF2α–GRP94 and IRE1α–XBP1–GRP78 arms of the endoplasmic reticulum stress response, collectively facilitating DNA damage repair and autophagy, which confer radio resistance to tumor cells [67]. Thus, XBP1-mediated radiotherapy resistance not only involves enhanced DNA repair capacity but is also closely associated with autophagic protection of tumor cells and remodeling of the inflammatory tumor microenvironment.

#### 3.3.4. Multifaceted Nature and Common Mechanisms of Therapy Resistance

Notably, XBP1-mediated therapy resistance is not confined to the individual mechanisms described above; rather, its effects span diverse therapeutic modalities and tumor types. In hepatocellular carcinoma, XBP1 upregulates IL-6 expression to activate the STAT3 signaling pathway, thereby promoting tumor cell proliferation and contributing to resistance to targeted therapies such as sorafenib [68]. In ovarian cancer, activation of the IRE1α–XBP1 pathway has been shown to be closely associated with cisplatin resistance, and pharmacological inhibition of IRE1α effectively reverses this resistance, restoring the chemosensitivity of tumor cells [69]. In melanoma, XBP1 sustains tumor cell viability by modulating MCL-1 expression and the endoplasmic reticulum stress response [34]. Collectively, these findings indicate that XBP1-mediated therapy resistance represents a highly conserved and multifaceted process, the core of which lies in helping tumor cells survive under various therapeutic pressures by regulating cellular metabolism, DNA damage repair, cell death pathways, and crosstalk with the tumor microenvironment. Consequently, as a central node connecting diverse stress signals with pro-survival pathways, XBP1 serves as a key driver of broad-spectrum therapy resistance in cancer.

In summary, whether in chemotherapy, endocrine therapy, or radiotherapy, activation of XBP1 constitutes a critical hub in the development of therapy resistance in tumor cells (Table 2). The underlying mechanisms are complex and diverse, ranging from post-transcriptional regulation and cell death pathways to signaling pathway crosstalk and microenvironment remodeling. A deeper understanding of these mechanisms will provide a solid theoretical foundation for the development of XBP1-targeted combination strategies aimed at overcoming tumor resistance.

## 4. XBP1 in the Tumor Microenvironment: Non-Autonomous Regulatory Roles

**Figure 3:**
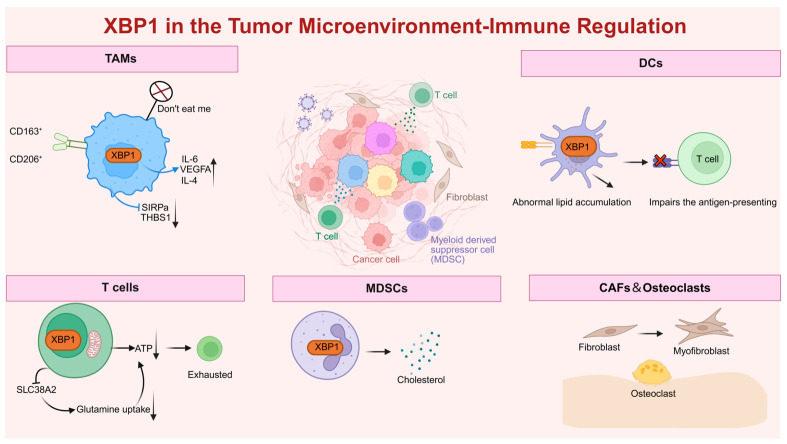
Activation of XBP1 in various cell types of the tumor microenvironment (TME) promotes immune evasion. In tumor-associated macrophages (TAMs), XBP1 induces M2 polarization (CD163^+^, CD206^+^), suppresses “don’t eat me” signals (SIRPα, THBS1), and upregulates pro-tumor cytokines (IL-6, VEGFA). In dendritic cells (DCs), XBP1 drives aberrant lipid accumulation, impairing antigen presentation and T cell priming. In T cells, XBP1 suppresses the glutamine transporter SLC38A2, leading to metabolic dysfunction and exhaustion. In myeloid-derived suppressor cells (MDSCs), tumor-derived, XBP1-dependent cholesterol-rich extracellular vesicles promote MDSC activation. XBP1 also contributes to cancer-associated fibroblast (CAF) activation and osteoclast-mediated bone resorption. Created in BioRender. Sun, H. (2026). https://BioRender.com/31txvgy.

The TME constitutes the “soil” on which tumor cells depend for survival, comprising various immune cells, stromal cells, vascular endothelial cells, and the extracellular matrix. Activation of XBP1 in these cell types profoundly influences antitumor immune responses. Therefore, a thorough understanding of the regulatory network governed by XBP1 within the TME is of great significance for the development of novel immunotherapeutic strategies (Figure 3).

### 4.1. Regulation of Tumor-Associated Macrophage (TAM) Function

Tumor-associated macrophages (TAMs) represent one of the most abundant immune cell populations in the TME. In solid tumors, TAMs typically exhibit an M2-polarized state, characterized by the expression of markers such as CD163 and CD206 and the secretion of anti-inflammatory cytokines including IL-10 and TGF-β, thereby promoting tumor growth, invasion, and metastasis. In recent years, accumulating evidence has established that XBP1, a key transcription factor in the ER stress response, plays a central role in shaping the pro-tumorigenic functions of TAMs. Indeed, XBP1 not only acts as a transcription factor but also serves as a critical hub linking tumor cell stress signals to the functional reprogramming of TAMs. Outside the tumor context, metabolic dysregulation in diabetes drives pulmonary injury through chronic inflammation, oxidative stress, and gut lung axis disruption, further supporting the concept that metabolic stress signals converge on transcription factors such as XBP1 to reprogram immune cell function [70].

#### 4.1.1. Maintenance of the Pro-Tumorigenic Phenotype of TAMs

In human colorectal cancer, infiltrating CD206^+^ TAMs exhibit specific activation of XBP1. Once activated, XBP1 upregulates the expression of pro-tumorigenic cytokines such as IL-6, VEGFA, and IL-4, thereby creating an inflammatory milieu conducive to tumor growth. More importantly, XBP1 activation directly suppresses the expression of SIRPα and THBS1, two key “don’t eat me” signaling molecules. Downregulation of these proteins blocks macrophage-mediated phagocytosis of tumor cells, thus synergistically promoting tumor growth and metastasis [20]. Collectively, these findings indicate that XBP1 serves as a critical molecular switch that sustains the immunosuppressive and pro-tumorigenic functions of TAMs, and further suggest that XBP1 represents a potential intervention target for reversing the pro-tumorigenic phenotype of TAMs.

#### 4.1.2. Promotion of M2 Polarization in Macrophages

Beyond its role in maintaining TAM functions, XBP1 is also essential for driving monocyte/macrophage polarization toward the M2 phenotype. Multiple tumor-derived signals induce this process by activating XBP1. For instance, exosomes derived from hepatocellular carcinoma cells transmit miR-21-5p to regulate the SP1/XBP1 signaling axis, thereby inducing M2 polarization of TAMs and accelerating liver cancer progression [71]. In addition, the environmental toxin microcystin-LR (MC-LR) activates the IRE1α/XBP1 pathway in colorectal cancer cells, promoting HK2 expression and lactate production; this metabolic reprogramming in turn induces M2 polarization of TAMs [72]. Similarly, in a glutamate-induced triple-negative breast cancer model, XBP1 activation mediates M2 polarization of macrophages, thereby facilitating tumor development [73]. Together, these studies reveal that XBP1 serves as a central hub linking tumor cell stress signals to the pro-tumorigenic polarization of TAMs, providing a critical target for interventions aimed at modulating TAM polarization. Macrophage metabolic reprogramming, including shifts in glutamine utilization and AMPK/PPARγ activity, is a central determinant of phenotypic polarization in muscle regeneration, further supporting the concept that XBP1 may control TAM function through analogous metabolic checkpoints [74].

#### 4.1.3. Regulation of the STING Signaling Pathway

The regulatory effect of XBP1 on TAM function also extends to its complex influence on the cGAS-STING innate immune signaling pathway. In the context of acute liver injury and liver fibrosis, XBP1-deficient hepatocytes or macrophages exhibit impaired mitophagy, leading to the release of mitochondrial DNA (mtDNA) into the cytoplasm. This, in turn, activates the cGAS-STING signaling pathway within macrophages, exacerbating inflammatory responses and fibrotic processes [3,9]. Of note, XBP1-mediated STING activation in macrophages also promotes liver fibrosis [9]. In the tumor context, STING activation has a dual nature. On the one hand, STING activation induces type I interferon production, thereby promoting antitumor immunity. On the other hand, persistent STING activation in specific microenvironments may lead to chronic inflammation and immunosuppression. Although direct evidence for XBP1 regulation of TAMs *via* STING signaling in the tumor setting remains insufficient, the aforementioned studies in liver fibrosis suggest that XBP1 may modulate mitochondrial homeostasis in TAMs, thereby influencing the intensity of STING signaling and ultimately playing a key role in tumor-associated inflammation and immune surveillance. A comprehensive review of the cGAS STING pathway in skeletal muscle pathophysiology similarly highlights its dual role—mediating both protective and pathological inflammation—and emphasizes the need for precise modulation to avoid unwanted toxicity, a lesson directly applicable to XBP1-based therapies in the TME [75]. Accordingly, in-depth investigation of the specific functions of the XBP1–STING signaling axis within the TME and its association with TAM polarization represents an important future research direction, with the potential to provide new insights for developing TAM-targeted cancer immunotherapies.

### 4.2. Impact on Dendritic Cell (DC) Function

Dendritic cells (DCs), as the most potent antigen-presenting cells in the body, play a central role in initiating and regulating T cell-mediated antitumor immune responses. However, within the TME, DC function is frequently suppressed, leading to the failure of antitumor immunity. Recent studies have identified the signaling pathway composed of the ER stress sensor IRE1α and its downstream transcription factor XBP1 as a critical node regulating DC function within the TME, where its aberrant activation contributes significantly to tumor immune evasion. Therefore, an in-depth understanding of the regulatory mechanisms governing the IRE1α-XBP1 axis in DCs holds substantial theoretical and clinical value for reversing the immunosuppressive state of the TME.

#### 4.2.1. Induction of Immunosuppressive DCs and Disruption of Their Metabolic Homeostasis

In various solid tumors, DCs within the TME undergo persistent and intense ER stress, leading to aberrant activation of the IRE1α-XBP1 pathway. This sustained activation profoundly reshapes DC metabolism and function. For instance, in ovarian cancer, continuous XBP1 activation in tumor-associated DCs (tDCs) transcriptionally upregulates triglyceride biosynthesis programs, resulting in abnormal lipid accumulation. This lipid metabolic disruption severely impairs the antigen-presenting capacity of DCs and compromises their ability to support antitumor T cell activation, thereby constituting a key mechanism of tumor immune evasion [9]. Furthermore, other TME factors, such as tumor-derived exosomes or free fatty acids, can exacerbate DC metabolic reprogramming and dysfunction by activating the IRE1α-XBP1 pathway [18]. Collectively, these findings reveal that XBP1-driven metabolic reprogramming is a core event underlying DC functional loss, underscoring its critical role in tumor immunosuppression.

Given the pivotal role of XBP1 in DCs, targeting this pathway has emerged as a potential strategy to restore antitumor immunity. Studies have demonstrated that specific knockout of XBP1 in DCs, or targeted silencing of XBP1 expression using nanoparticles, effectively restores the immunostimulatory activity of DCs and reverses their immunosuppressive phenotype. Consequently, this induces robust type I antitumor immune responses and significantly prolongs the survival of tumor-bearing mice [21]. Thus, intervening in the XBP1 signaling pathway in DCs holds promise for relieving the TME-induced “blockade” on DCs, reactivating effective antitumor immune responses, and providing a novel entry point for cancer immunotherapy.

#### 4.2.2. Suppression of NKG2D Ligand Expression to Evade Innate Immune Killing

Beyond affecting DC function, XBP1 activation in tumor cells themselves also contributes to immune evasion. Activation of the XBP1 pathway in tumor cells helps them evade surveillance by natural killer (NK) cells. Specifically, activation of the IRE1α-XBP1 axis in tumor cells suppresses the expression of major histocompatibility complex class I chain-related A (MICA), a ligand for the NK cell activating receptor NKG2D, through the regulation of the transcription factor E2F1 [76]. Downregulation or loss of MICA on the tumor cell surface enables tumor cells to mask themselves from NK cell recognition and killing, thereby gaining a survival advantage. This mechanism reveals that, in addition to promoting its own survival and proliferation, XBP1 in tumor cells actively facilitates evasion of innate immune attack by downregulating immunorecognition molecules. Consequently, XBP1 activation in tumor cells represents another critical route for evading innate immune surveillance, further solidifying its potential as a target for immunotherapy.

In summary, XBP1 exerts a dual immunosuppressive role within the tumor immune microenvironment. On one hand, its aberrant activation in DCs impairs their antigen-presenting function by disrupting lipid metabolism, thereby inhibiting the initiation of adaptive immune responses. On the other hand, its activation in tumor cells helps these cells evade NK cell-mediated innate immune surveillance by downregulating NKG2D ligand expression. Therefore, the XBP1 signaling pathway is not only a key driver of tumor cell survival but also a central hub that reshapes the entire immune microenvironment to facilitate immune evasion. A deep understanding of the regulatory networks of XBP1 in distinct immune cell types and tumor cells will provide a solid theoretical foundation and novel intervention targets for developing combination immunotherapeutic strategies aimed at this pathway.

### 4.3. Regulation of T Cell Function and Exhaustion

As the core effector cells in antitumor immune responses, T cells must function properly to eliminate tumor cells. However, in both solid tumors and hematological malignancies, metabolic reprogramming and harsh conditions within the TME—such as hypoxia, nutrient deprivation, and acidic pH—frequently impair the function of infiltrating T cells and drive them toward exhaustion. Recent studies have identified the key endoplasmic reticulum stress regulator XBP1 as a central player in T cell metabolic adaptation and functional regulation. Nevertheless, its role in the tumor context is complex: XBP1 can both drive T cell dysfunction and, under specific conditions, exert protective effects. Therefore, a thorough understanding of the functional heterogeneity of XBP1 across different tumor types and immune cell subsets is essential for developing precise and effective immunotherapeutic strategies.

#### 4.3.1. Induction of Metabolic Dysfunction and Exhaustion in T Cells

Within the TME—particularly under extreme conditions such as malignant ascites—T cells experience profound metabolic stress. Accumulating evidence indicates that the IRE1α–XBP1 signaling axis is a critical link connecting T cell metabolic stress to functional inhibition. In ovarian cancer, for example, lipid- and cytokine-rich malignant ascites suppresses glucose uptake in T cells and induces defects in N-linked protein glycosylation, thereby activating the IRE1α–XBP1 pathway. Once activated, XBP1 directly represses the expression of glutamine transporters, limiting glutamine uptake by T cells. Given that, under glucose-deprived conditions, glutamine serves as a key alternative fuel for maintaining mitochondrial respiration and producing interferon-γ (IFN-γ) in T cells, activation of XBP1 severely impairs both mitochondrial respiratory capacity and effector function, ultimately compromising antitumor activity [77]. Similarly, in multiple myeloma, XBP1 suppresses expression of the glutamine transporter SLC38A2, leading to reduced glutamine uptake and consequent immune dysfunction in T cells [78]. Collectively, these findings identify XBP1 as a critical metabolic checkpoint that limits T cell effector function across multiple tumor types by negatively regulating glutamine metabolism. In summary, XBP1-mediated metabolic reprogramming represents a core mechanism underlying the functional exhaustion of tumor-infiltrating T cells.

#### 4.3.2. Regulation of T Cell Survival and Differentiation

Notably, the regulation of T cell function by XBP1 is not invariably negative; rather, its effects are highly dependent on cell type and disease context. In several tumor models, targeted inhibition of XBP1 or its upstream regulator IRE1α effectively restores T cell antitumor activity. For instance, in multiple myeloma, the XBP1–SLC38A2 axis has been identified as a key regulator of T cell metabolism, and targeting this axis holds promise for reversing T cell exhaustion [78]. In acute myeloid leukemia (AML), however, XBP1 appears to play a protective role. Studies have shown that, in hematopoietic stem and progenitor cells, XBP1 maintains hematopoietic integrity by suppressing pro-leukemic gene programs. Specifically, loss or inactivation of XBP1 relieves the inhibition of oncogenic pathways such as Wnt–β-catenin signaling, thereby promoting leukemogenesis [42]. This indicates that, in the context of AML, XBP1 serves a critical “gatekeeper” function in preserving normal hematopoietic stem cell function and preventing malignant transformation, with its absence actually accelerating disease progression. Thus, the impact of XBP1 on T cells and their precursors is highly context-dependent, underscoring that the development of XBP1-targeted therapeutic strategies must carefully account for this potential cell type- and disease context-specificity.

In conclusion, XBP1 plays a dual role in regulating T cell function and exhaustion. On the one hand, within the solid tumor microenvironment, aberrant activation of XBP1 represents a major mechanism driving metabolic dysfunction and exhaustion in T cells. On the other hand, in hematological malignancies, XBP1 is essential for maintaining normal hematopoietic stem cell function. These findings not only deepen our understanding of tumor immune evasion mechanisms but also highlight the need for precise and careful modulation when targeting XBP1 therapeutically, so as to effectively reverse T cell exhaustion in the tumor microenvironment without compromising normal immune function. Future research should focus on exploring how to achieve precise immune interventions by modulating XBP1 activity according to specific tumor types and immune contexts, thereby translating this complex molecular target into clinically effective treatment strategies.

### 4.4. Regulation of Other Tumor Microenvironment Components

The TME constitutes a complex ecosystem comprising multiple cell types, wherein interactions among tumor cells, stromal cells, and immune cells profoundly influence tumor progression, metastasis, and therapeutic resistance. In addition to the aforementioned tumor-associated macrophages and T cells, the XBP1 signaling pathway plays a critical role in reshaping other key components of the TME, as detailed below.

#### 4.4.1. Myeloid-Derived Suppressor Cells (MDSCs)

MDSCs represent a major immunosuppressive cell population within the TME, capable of suppressing the antitumor functions of T cells. Notably, cancer cell-intrinsic XBP1 signaling plays a pivotal role in driving the immunosuppressive function of MDSCs. Studies have demonstrated that XBP1 in cancer cells promotes cholesterol synthesis and secretion, packaging cholesterol into small extracellular vesicles that are subsequently transferred to MDSCs. These vesicles are taken up by MDSCs *via* macropinocytosis, leading to elevated intracellular cholesterol levels. This, in turn, activates MDSCs and induces a potent immunosuppressive phenotype, ultimately facilitating tumor immune evasion [18]. This discovery reveals a novel mechanism by which tumor cells, through XBP1-mediated metabolic reprogramming, “educate” MDSCs in a non-cell-autonomous manner to establish an immunosuppressive microenvironment, thereby providing a theoretical basis for targeting this pathway to relieve immunosuppression.

#### 4.4.2. Cancer-Associated Fibroblasts (CAFs)

Fibroblast activation and transdifferentiation represent core events in tumor fibrosis and matrix remodeling. Indeed, XBP1 has been established as a key driver of fibroblast activation in various non-neoplastic diseases. For instance, in the context of liver fibrosis and nonalcoholic steatohepatitis (NASH), activation of XBP1 in macrophages promotes the secretion of transforming growth factor-beta 1 (TGF-β1), which in turn activates hepatic stellate cells (HSCs) and drives their transdifferentiation into myofibroblasts, thereby exacerbating liver fibrosis [79]. Moreover, XBP1 itself can directly enhance the fibrotic activity of HSCs by inducing autophagy, thus creating a positive feedback loop that amplifies the fibrotic response [80]. Similarly, transcriptome profiling of angiotensin II induced cardiac hypertrophy revealed that inflammatory and fibrotic gene upregulation drives pathological remodeling, suggesting that ER stress associated transcription factors such as XBP1 could participate in analogous stromal reprogramming in the tumor microenvironment [81]. In triple-negative breast cancer (TNBC), IRE1α disruption significantly remodels the cellular tumor microenvironment, notably decreasing the abundance of cancer-associated fibroblasts alongside myeloid-derived suppressor cells [82], indicating that tumor-cell-intrinsic IRE1α–XBP1 signaling is required for CAF accumulation in the TME. Additionally, XBP1s overexpression has been shown to induce de-differentiation of mammary adipocytes into fibroblast-like precursor cells that integrate into the tumor microenvironment and promote tumor progression [83], suggesting a broader role for XBP1 in generating CAF-like cells from adjacent stromal progenitors. Although this evidence is derived primarily from liver fibrosis models, given the functional similarities between CAFs and activated HSCs, it is plausible that XBP1 signaling originating from tumor cells or macrophages in the TME may similarly induce CAF activation, thereby promoting tumor progression and matrix remodeling. This opens new perspectives for understanding tumor–stroma interactions.

#### 4.4.3. Osteoclasts

In multiple myeloma (MM), a hematological malignancy characterized by osteolytic lesions, the XBP1 signaling pathway plays a critical role in osteoclast differentiation. It has been demonstrated that small extracellular vesicles derived from myeloma cells are taken up by osteoclast precursors, activating the IRE1α/XBP1 signaling axis within these cells. XBP1 activation subsequently promotes osteoclast differentiation and maturation, enhancing bone resorption activity and ultimately exacerbating osteolytic lesions in patients with multiple myeloma [42]. Mechanistically, MM-derived small extracellular vesicles (MM-EVs) induce rapid phosphorylation of IRE1α at Ser724 and increase *Xbp1* mRNA splicing in murine macrophages, which in turn activates the transcription of NFATc1, a master transcription factor essential for osteoclast differentiation [84]. Beyond the direct effects of MM-EVs on osteoclast precursors, XBP1 signaling in bone marrow stromal cells (BMSCs) within the MM microenvironment also critically contributes to osteoclast formation. XBP1s is induced in BMSCs of the MM microenvironment, and its overexpression in healthy human BMSCs enhances the gene and/or protein expression of VCAM-1, IL-6, and receptor activator of NF-κB ligand (RANKL), thereby promoting both MM cell growth and osteoclast formation in vitro and in vivo [85]. Conversely, knockdown of XBP1 in MM patient BMSCs reverses these effects, compromising their enhanced support of MM cell growth and osteoclast formation in response to TNFα stimulation [85]. Thus, XBP1s functions as a pathogenic factor in BMSCs that drives osteoclastogenesis through paracrine mechanisms involving RANKL and IL-6 secretion. This finding not only elucidates how myeloma cells “co-opt” osteoclasts to remodel the bone microenvironment but also highlights the XBP1 signaling axis as a critical node linking tumor cells and the bone microenvironment, offering a potential intervention target for treating MM-associated bone disease.

#### 4.4.4. Endothelial-Cells

Tumor angiogenesis, the process by which new blood vessels form from pre-existing vasculature, is essential for tumor growth, invasion, and metastasis. XBP1 has emerged as a critical regulator of angiogenesis through both direct effects on endothelial cells and indirect modulation of pro-angiogenic factors.

Direct evidence for XBP1-mediated regulation of endothelial cell function comes from multiple studies. In lung cancer, knockdown of the stress response molecule NUPR1 inhibits angiogenesis through the IRE1/XBP1 and PERK/eIF2α/ATF4 signaling pathways, resulting in reduced VEGFA expression and impaired endothelial cell tube formation and migration [86]. This demonstrates that XBP1 signaling in tumor cells directly influences the angiogenic capacity of endothelial cells via paracrine mechanisms. In glioblastoma, protein disulfide-isomerase A4 (PDIA4) is upregulated by ER stress through transcriptional regulation by XBP1, forming an XBP1/PDIA4/VEGFA axis that promotes GBM angiogenesis both in vitro and in vivo [87]. Importantly, GBM cells with high PDIA4 expression exhibit resistance to anti-angiogenic therapy, suggesting that XBP1-driven PDIA4 upregulation contributes to therapeutic resistance [87]. In triple-negative breast cancer, IRE1α disruption reverses ER stress adaptation and significantly remodels the tumor microenvironment, normalizing tumor vasculature and synergizing with anti-VEGFA treatment to cause tumor stasis or regression [82]. Pharmacologic IRE1α kinase inhibition not only attenuates tumor growth but also increases pericyte numbers while decreasing cancer-associated fibroblasts and myeloid-derived suppressor cells, indicating that XBP1 signaling broadly coordinates angiogenesis with immune and stromal remodeling [82].

Beyond these direct mechanisms, XBP1 also influences angiogenesis through regulation of additional factors. In inflammatory breast cancer cells, ER stress activation modulates the expression of UPR components including XBP1, and manipulation of ER stress with Salubrinal induces cancer cell death while affecting angiogenic signaling [88]. Furthermore, XBP1 has been shown to transcriptionally regulate VEGFA expression through cooperation with HIF1α under hypoxic conditions, and both the UPR and hypoxia pathways influence VEGF expression by increasing the transcription factors ATF4 and XBP1 [89]. The IRE1 branch of the UPR increases HIF stabilization via regulated IRE1-dependent decay (RIDD), further linking XBP1 signaling to hypoxia-driven angiogenesis [89].

Collectively, these findings establish XBP1 as a central integrator of angiogenesis signals in the tumor microenvironment, operating through multiple interconnected mechanisms. Targeting the XBP1 pathway therefore represents a promising strategy to enhance the efficacy of anti-angiogenic therapies across multiple cancer types.

In summary, the role of XBP1 in the TME extends far beyond the regulation of classical immune cells. Through its modulation of metabolic reprogramming in MDSCs, activation of CAFs, differentiation of osteoclasts, and endothelial cell function, XBP1 broadly participates in multiple key processes that drive tumor malignancy, including immunosuppression, matrix remodeling, bone destruction, and vascularization. Collectively, these findings paint a comprehensive picture of XBP1 as a central regulator of the TME and underscore the tremendous potential of targeting the XBP1 signaling pathway in the development of integrated cancer therapeutic strategies.

## 5. The Complexity and Bidirectional Regulation of the XBP1 Signaling Pathway

**Figure 4:**
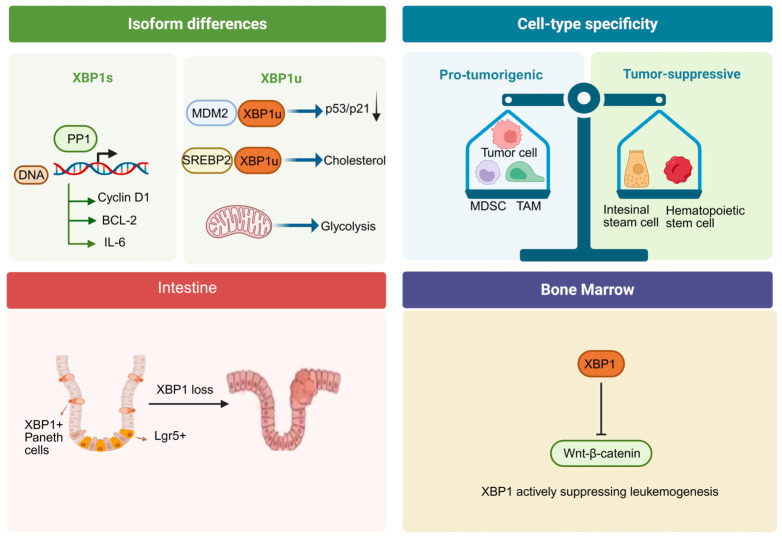
XBP1 exhibits cell-type- and context-dependent functions, acting either as a pro-tumorigenic or tumor-suppressive factor. In cancer cells and pro-tumor immune cells (MDSCs, TAMs), XBP1 (predominantly XBP1s) drives the expression of Cyclin D1, BCL-2, IL-6, and genes involved in cholesterol metabolism. In contrast, XBP1u can interact with MDM2 to enhance its stability, thereby negatively regulating the p53/p21 tumor suppressor axis and promoting cell cycle progression. In intestinal stem cells and hematopoietic stem cells, XBP1 plays a protective role: loss of XBP1 leads to aberrant Lgr5^+^ stem cell expansion and spontaneous intestinal tumorigenesis, while in hematopoietic progenitors the IRE1α–XBP1 axis restricts pro-leukemogenic programs (e.g., Wnt–β-catenin signaling) and suppresses acute myeloid leukemia development. Created in BioRender. Sun, H. (2026). https://BioRender.com/iofktfp.

The role of XBP1 in tumor development is not simply “pro-tumorigenic” or “tumor-suppressive”; instead, it exhibits remarkable complexity and bidirectionality. This functional duality depends on cell type, the tumor microenvironment, stress intensity, and the specific molecular context. In recent years, accumulating evidence has revealed that the regulatory network of XBP1 is far more sophisticated than originally envisioned, and its role in tumor biology requires a more dynamic and multi-dimensional interpretation. Therefore, a thorough understanding of the mechanisms underlying the functional duality of XBP1 is essential for dissecting tumor biology and developing precision therapeutic strategies (Figure 4).

### 5.1. Functional Differences Between XBP1u and XBP1s

For a long time, XBP1u was primarily regarded as an inactive precursor of the XBP1s. However, recent studies have thoroughly overturned this traditional view, revealing that XBP1u exerts independent and critical functions in tumorigenesis that do not rely on XBP1s. For example, XBP1u negatively regulates the canonical p53/p21 tumor suppressor axis by interacting with MDM2 and enhancing its stability, thereby promoting cell cycle progression and tumorigenesis [90]. In hepatocellular carcinoma, XBP1u enhances cholesterol biosynthesis by stabilizing SREBP2, a key transcription factor in cholesterol synthesis, thereby providing essential lipid substrates for rapid tumor cell proliferation and driving tumorigenesis [44]. Furthermore, XBP1u can interfere with the mitochondrial localization of the mitochondrial genome maintenance exonuclease MGME1, reducing mitochondrial DNA copy number and mitochondrial abundance, rewiring the metabolic pattern of tumor cells towards aerobic glycolysis (the Warburg effect), and consequently promoting colorectal cancer progression [55]. Collectively, these findings indicate that the two XBP1 isoforms play synergistic but distinct roles in tumors: XBP1s primarily functions as a transcription factor directly regulating downstream effector genes, whereas XBP1u modulates key signalling pathways at the post-translational level through protein–protein interactions. Together, they constitute a complex and finely tuned regulatory network. Therefore, comprehensively dissecting the synergistic and antagonistic mechanisms of XBP1u and XBP1s in specific tumor types represents a central aspect of understanding the functional duality of XBP1.

### 5.2. Differential Effects in Distinct Cell Types and Tissue Environments

The function of XBP1 is highly cell-type- and tissue-specific, a feature that is particularly evident during the progression of non-alcoholic steatohepatitis (NASH). Studies have shown that in mouse models of NASH, hepatocyte-specific *Xbp1* knockout suppresses NASH progression primarily by reducing lipid accumulation; conversely, macrophage-specific *Xbp1* knockout alleviates NASH and liver fibrosis by inhibiting inflammatory responses [42]. These findings suggest that XBP1 promotes disease progression in a coordinated manner through distinct mechanisms in different cell types within the liver.

The role of XBP1 in immune cells is similarly complex. In ovarian cancer, sustained endoplasmic reticulum stress induced by the tumor microenvironment activates XBP1 in tumor-infiltrating dendritic cells, leading to aberrant lipid metabolism and impaired immunostimulatory function, thereby suppressing anti-tumor T cell activation and promoting immune evasion [21]. In contrast, activation of the IRE1α–XBP1 axis in T cells is crucial for mitochondrial function and effector capacity; however, in the hostile microenvironment of ovarian cancer, persistent XBP1 activation paradoxically suppresses T cell metabolic fitness and effector function [77]. This dual role of XBP1—both protective and destructive—within immune cells highlights its complex position in tumor immune surveillance. A similar duality exists for macrophages in skeletal muscle, where proper M1/M2 polarization is required for regeneration, but persistent M1 dominance drives atrophy through NF κB and JAK STAT activation—underscoring that the functional outcome of immune cell activation is highly context dependent, as seen for XBP1 in the TME [91].

Notably, high XBP1 expression in lung cancer is associated with a favourable prognosis. Single-cell analyses have revealed that this may be attributable to XBP1 promoting the adaptive survival of plasma cells within the tumor microenvironment, potentially exerting anti-tumor effects—a striking contrast to the pro-malignant role of XBP1 in most other cancers [26]. This finding strongly suggests that the prognostic significance and functional role of XBP1 are highly tissue-specific, and simply categorising XBP1 as a “pro-tumorigenic” or “tumor-suppressive” gene is an oversimplification. Collectively, these observations underscore that the functional interpretation of XBP1 must be contextualised within specific cell types and microenvironments, and any generalisation that disregards the specific context may lead to erroneous conclusions.

### 5.3. The “Double-Edged Sword” Effect of XBP1 in Maintaining Normal Tissue Homeostasis and Tumorigenesis

XBP1 also plays a critical role in maintaining stem cell homeostasis in normal tissues, and its loss of function can sometimes be even more tumorigenic than its gain of function. In the intestine, XBP1 is a key factor in sustaining intestinal stem cell (ISC) homeostasis and epithelial barrier integrity. Deletion of *Xbp1* induces an IRE1α-dependent regenerative response, leading to aberrant expansion of Lgr5^+^ ISCs and increased susceptibility to colitis-associated and spontaneous intestinal tumorigenesis [92]. This finding stands in stark contrast to the pro-tumorigenic role of XBP1 in most solid tumors, where it promotes cancer cell proliferation, survival, and metastasis. Such context-dependent functionality—tumor-suppressive in normal tissues but tumor-promotive in established malignancies—highlights the functional switch of XBP1 under different pathophysiological settings and explains why XBP1 expression correlates with favorable prognosis in some cancer types. Moreover, XBP1 exerts a protective role in the hematopoietic system. Activation of the IRE1α-XBP1 axis has been shown to suppress pro-leukemogenic gene programs (e.g., the Wnt-β-catenin pathway) in hematopoietic stem and progenitor cells, thereby protecting them from malignant transformation and suppressing the development of acute myeloid leukemia [42]. These observations further demonstrate that the XBP1 pathway can act as a critical molecular switch governing cell fate decisions among differentiation, homeostasis maintenance, and malignant transformation, depending on the tissue type and physiological or pathological state. Collectively, these findings reveal that XBP1 serves a dual role as both a key “homeostasis guardian” in normal physiology and a “driver of malignant transformation” in cancer.

In summary, the functions of the XBP1 signaling pathway in cancer are far from linear or unidirectional regulation; instead, they constitute a complex network that integrates information on cell type, stress signals, and molecular context. The functional divergence between XBP1u and XBP1s, their differential roles across immune cell types and tissues, and their essential involvement in normal stem cell homeostasis collectively underpin the bidirectionality and complexity of XBP1 function. Therefore, when developing therapeutic strategies targeting XBP1, this complexity must be thoroughly considered to suppress tumors while minimizing interference with normal tissue homeostasis and avoiding potential pro-tumorigenic risks. Future research should focus on elucidating the molecular switches that govern the specific functions of XBP1 across distinct cell lineages and under various stress states, thereby laying a solid foundation for the development of more selective and safer targeted therapies.

## 6. Therapeutic Strategies Targeting XBP1 and Associated Challenges

**Figure 5:**
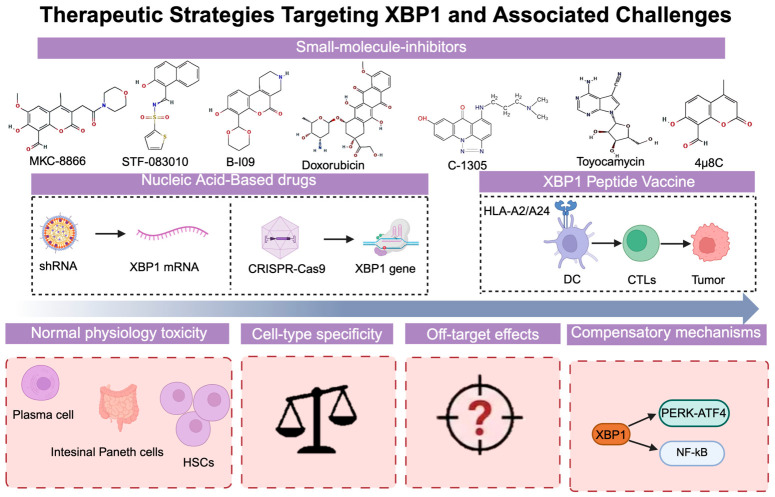
Multiple therapeutic strategies targeting the IRE1α–XBP1 pathway have been developed, including small-molecule inhibitors (MKC-8866, STF-083010, toyocamycin, B-I09, 4μ8C), nucleic acid-based agents (siRNA, shRNA, CRISPR-Cas9), and XBP1 peptide vaccines. These approaches have shown preclinical efficacy in inhibiting tumor growth, overcoming therapy resistance, and enhancing antitumor immunity. However, major challenges remain: (1) on-target toxicity due to the essential roles of XBP1 in normal tissues (e.g., plasma cells, Paneth cells, hematopoietic stem cells); (2) cell-type-specific and context-dependent functions; (3) limited selectivity and off-target effects of current inhibitors; and (4) compensatory activation of other UPR branches (e.g., the PERK–ATF4 pathway) that may drive adaptive resistance. Created in BioRender. Sun, H. (2026). https://BioRender.com/5ix5dti.

Given the central role of XBP1 in tumorigenesis, metabolic reprogramming, and immune evasion, targeting its upstream activator IRE1α or directly interfering with XBP1 activity has emerged as an attractive anticancer strategy (Figure 5).

### 6.1. Small-Molecule Inhibitors

Given the critical functions of the IRE1α–XBP1 signaling axis in tumor initiation, progression, drug resistance, and immune evasion, the development of small-molecule inhibitors targeting this pathway has become a major focus of cancer research. To date, several such inhibitors have been developed, primarily targeting either the kinase or the RNase activity of IRE1α, thereby blocking XBP1 splicing and activation, suppressing tumor cell survival, and enhancing sensitivity to other therapies.

#### 6.1.1. Inhibitors of IRE1α RNase Activity

The RNase activity of IRE1α is essential for generating the functional transcription factor sXBP1; therefore, directly inhibiting this activity represents the most direct strategy to block the pathway. In recent years, a series of structurally diverse RNase inhibitors have been reported and have demonstrated promising antitumor efficacy in various preclinical tumor models. Among these, MKC-8866 is one of the most commonly used IRE1α RNase inhibitors in preclinical studies, showing marked antitumor effects across multiple tumor models through various mechanisms, including synthetic lethality or synergistic interactions depending on the tumor context. For example, in MYC-driven breast cancer, MKC-8866 exhibits synthetic lethality, suggesting its specific potential to kill tumors with high MYC expression [58]. Furthermore, this inhibitor enhances the chemosensitivity of glioblastoma to temozolomide [93] and effectively suppresses the growth of prostate cancer [7] and rhabdomyosarcoma [94]. Collectively, these findings indicate that MKC-8866 is a promising IRE1α inhibitor, particularly suitable for combination with other therapies to overcome drug resistance and enhance therapeutic efficacy.

Similar to MKC-8866, STF-083010, an inhibitor that specifically blocks XBP1 splicing, has shown significant value in reversing endocrine resistance. In estrogen receptor-positive (ER^+^) breast cancer, it effectively reverses tamoxifen resistance, suggesting that it may exert therapeutic effects by restoring the sensitivity of ER^+^ breast cancer cells to endocrine therapy [95]. This finding provides a new strategy to overcome clinically common endocrine therapy resistance and highlights the therapeutic potential of targeting XBP1 splicing in hormone-dependent tumors. Toyocamycin, identified as an IRE1α–XBP1 pathway inhibitor from microbial cultures [96], exhibits antitumor activity in both solid and hematological malignancies. Studies have confirmed its ability to suppress tumor growth in Ewing sarcoma [97] and multiple myeloma [96], indicating its potential as a broad-spectrum anticancer agent and warranting further preclinical evaluation across additional tumor types. Moreover, B-I09 has shown promise in tumors with specific genetic backgrounds. In ARID1A-mutated ovarian clear cell carcinoma, B-I09 effectively inhibits tumor growth [98]. Given that ARID1A is a subunit of the SWI/SNF chromatin remodeling complex and its mutations are prevalent across multiple cancer types, B-I09 may offer a new targeted therapeutic option for these difficult-to-treat tumors harboring specific gene mutations, enabling precision therapy.

In addition to the inhibitors mentioned above, 4μ8C, which also inhibits IRE1α RNase activity, has shown therapeutic potential in various disease models. In cancer-related studies, it not only suppresses cell proliferation and invasion in colorectal cancer [98] but also attenuates muscle wasting in a pancreatic cancer cachexia model [37], suggesting potential value in alleviating tumor-associated complications. Furthermore, 4μ8C has been widely used to investigate the role of IRE1α in non-neoplastic diseases, such as liver fibrosis [9] and inflammatory responses [99], serving as an important tool compound for exploring the physiological and pathological functions of the IRE1α signaling pathway. In contrast, 9-*N*-[3-(dimethylamino)propyl]-3-*N*,3-*N*,6-*N*,6-*N*-tetramethylacridine-3,6,9-triamine (3,6-DMAD), a derivative of *N*-acridin-9-yl-*N*’,*N*’-dimethylpropane-1,3-diamine (DAPA), has a distinct mechanism of action: it simultaneously inhibits both the oligomerization and endonuclease activities of IRE1α, thereby more completely blocking XBP1 activation. Studies have shown that this compound exerts significant cytotoxicity against multiple myeloma cells [100]. Its dual inhibitory mechanism may provide a solution to overcome resistance associated with single-mechanism inhibitors and holds promise for the development of more potent anticancer agents.

#### 6.1.2. Inhibitors of IRE1α Kinase Activity

The kinase activity of IRE1α is a prerequisite for its autophosphorylation and subsequent activation of its RNase activity. Consequently, small-molecule compounds targeting its kinase activity can indirectly inhibit XBP1 splicing.

The development of selective IRE1α kinase inhibitors has progressed more slowly, largely due to the high conservation of the kinase ATP-binding pocket across the kinome, which poses challenges for achieving selectivity. Early studies relied on multi-kinase inhibitors that non-selectively suppressed IRE1α phosphorylation, but their off-target effects limited their utility for mechanistic dissection. More recently, structure-guided approaches have led to the discovery of ATP-competitive inhibitors of IRE1α with improved selectivity, which bind to the kinase domain and allosterically or directly block RNase activation. Several representative kinase-targeting compounds have been evaluated in cancer models. APY29 is a selective ATP-competitive IRE1α kinase inhibitor that suppresses autophosphorylation and downstream XBP1 splicing. In TNBC, APY29 treatment unexpectedly upregulates the ferroptosis suppressor xCT (SLC7A11) and promotes glutathione synthesis, thereby increasing resistance to ferroptosis [101]. This finding reveals a context-dependent, pro-survival role of IRE1α kinase activity and cautions that kinase inhibition may produce opposite effects in different cellular settings. KIRA6 is another ATP-competitive IRE1α kinase inhibitor that blocks both kinase and RNase functions. Encapsulation of KIRA6 in a redox-sensitive nanoemulsion (α-T-K) effectively repolarized M2-polarized tumor-associated macrophages under hypoxic conditions, delayed tumor growth, and sensitized anti-PD-1 immunotherapy [102]. Nevertheless, the structural diversity of IRE1α kinase inhibitors remains limited, and systematic structure–activity relationship (SAR) studies are still in their infancy.

The selective inhibitors directly targeting IRE1α kinase activity often face challenges including low selectivity and a high risk of off-target effects. With an increasing understanding of the IRE1α kinase domain, the development of highly selective kinase inhibitors remains an important future research direction, offering potential for combination with other kinase inhibitors or cytotoxic drugs. For instance, doxorubicin has been found to inhibit XBP1 activation independently of its topoisomerase II inhibitory effects, suggesting that it may exert part of its anticancer effects by influencing IRE1α kinase activity [103]. Similarly, triazoloacridine C-1305 has been shown to impair XBP1 splicing by inhibiting the endonuclease activity of IRE1α [104]. In summary, pharmacological modulation of IRE1α kinase activity offers a distinct strategy to suppress the IRE1α-XBP1 axis, but current inhibitors face challenges of selectivity, context-dependent effects, and potential pro-survival outcomes. Future medicinal chemistry efforts should focus on developing isoform-selective, ATP-competitive or allosteric IRE1α kinase inhibitors with well-defined SAR, and systematically evaluating their antitumor efficacy alone or in combination with chemotherapy and immunotherapy.

### 6.2. Nucleic Acid-Based Drugs and Gene Editing

Given the central role of XBP1 in tumor initiation, progression, metastasis, immune evasion, and drug resistance, developing therapeutic strategies targeting its expression or function has emerged as a highly promising research direction [105]. Currently, various nucleic acid-based drugs and gene-editing technologies are being employed to precisely regulate XBP1 expression, demonstrating significant antitumor potential in preclinical models.

#### 6.2.1. siRNA/shRNA-Mediated Gene Silencing

RNA interference (RNAi)-mediated silencing of XBP1 represents one of the most widely applied strategies. Delivering small interfering RNA (siRNA) or short hairpin RNA (shRNA) into tumor cells or key immune cells within the tumor microenvironment can effectively block the XBP1 signaling pathway. For example, aptamer-conjugated RNA nanoparticle systems delivering XBP1 siRNA can specifically target HER2-positive breast cancer cells, not only effectively inhibiting tumor growth but also enhancing sensitivity to chemotherapeutic agents [106]. Furthermore, lentivirus-mediated XBP1 shRNA has demonstrated significant antitumor effects in colorectal cancer and glioma models, with mechanisms closely linked to the inhibition of tumor cell proliferation, induction of apoptosis, and regulation of metabolic reprogramming (e.g., suppression of the glycolytic key enzyme HK2) [107,108]. Collectively, these studies confirm that directly silencing XBP1 in tumor cells is an effective therapeutic strategy, laying a foundation for subsequent clinical translation.

#### 6.2.2. CRISPR-Cas9 Gene Editing

Compared with transient RNAi-mediated silencing, CRISPR-Cas9 technology enables permanent knockout of the XBP1 gene, thereby offering a more definitive therapeutic strategy. For instance, using an adeno-associated virus (AAV) to deliver the CRISPR-Cas9 system for specific knockout of XBP1 in tumor-associated macrophages (TAMs) within colorectal cancer models significantly suppressed tumor progression [20]. This study further revealed that XBP1 knockout in macrophages blocks “don’t eat me” signals, enhances macrophage phagocytosis of tumor cells, and inhibits the secretion of protumor cytokines, thereby remodeling the antitumor immune microenvironment [20]. In addition, AAV2-mediated delivery of sgXBP1 for gene editing has also validated its antitumor activity in colorectal cancer models [20]. These findings collectively demonstrate the feasibility and efficacy of targeting XBP1 in TAMs via gene-editing approaches, providing new avenues for the development of novel immunotherapies.

In summary, from RNAi to CRISPR-Cas9, multiple nucleic acid-based drugs and gene-editing strategies have been successfully employed to target XBP1, all demonstrating potent antitumor activity in preclinical models. Future research efforts should focus on optimizing the delivery systems for these nucleic acid agents, improving their tumor targeting, bioavailability, and safety, with the goal of translating these promising strategies into clinical applications and bringing new treatment options to cancer patients. Therefore, therapeutic strategies targeting XBP1 not only hold potential for overcoming resistance to existing therapies but may also enhance the efficacy of immunotherapy by remodeling the tumor microenvironment.

### 6.3. Combination Immunotherapy Strategies

Given the central role of XBP1 in reshaping the immunosuppressive tumor microenvironment, its combination with immunotherapies aims to release the “brake” imposed on the immune system, thereby demonstrating substantial synergistic therapeutic potential. Current strategies primarily focus on the following two aspects.

#### 6.3.1. Enhancing the Efficacy of Immune Checkpoint Inhibitors

Tumor cells exploit the IRE1α-XBP1 pathway, activated by endoplasmic reticulum stress, to functionally reprogram various immune cells, thereby constructing a robust immunological barrier. Consequently, inhibiting this pathway holds promise for reversing immunosuppression at multiple levels and sensitizing tumors to immune checkpoint inhibitors (ICIs). First, at the level of myeloid-derived suppressor cells (MDSCs), studies have demonstrated that tumor cells significantly recruit and activate MDSCs through XBP1-dependent cholesterol synthesis and secretion. Conversely, inhibiting the IRE1α-XBP1 pathway effectively reduces MDSC infiltration and activation, thereby alleviating their suppression of T cells [18]. Second, at the dendritic cell (DC) level, lipid peroxides in the tumor microenvironment persistently activate XBP1 in DCs, inducing aberrant lipid accumulation that impairs their antigen-presenting function. Targeting XBP1 can restore the immunostimulatory capacity of DCs and promote the priming of antitumor T cells [21]. Finally, at the T cell level, metabolic stress (e.g., glucose deprivation) within the tumor microenvironment activates IRE1α-XBP1 signaling in infiltrating T cells. This signaling limits their mitochondrial respiration by suppressing the expression of the glutamine transporter SLC38A2, ultimately leading to T cell exhaustion and dysfunction [109]. In summary, reversing T cell exhaustion by interfering with the IRE1α-XBP1 pathway is a critical step in restoring the efficacy of ICIs. Furthermore, combining this approach with other targeted therapies has shown synergistic effects. For instance, in c-Myc-driven aggressive B-cell lymphomas, elevated endoplasmic reticulum stress resulting from high c-Myc expression renders tumor cells more sensitive to XBP1 inhibitors. Research confirms that combining an XBP1 inhibitor with a PARP inhibitor produces synergistic cytotoxicity, offering a novel therapeutic strategy for such refractory tumors [110]. Notably, restoring gut microbial and metabolic homeostasis with natural compounds such as Tectorigenin alleviates diabetic lung injury by suppressing macrophage M1 polarization and oxidative stress via the Nrf2/GPX1 axis, illustrating how metabolic interventions could complement XBP1 targeted strategies to remodel the immune microenvironment [111]. This finding underscores the broad potential of combining XBP1 targeting with DNA damage repair pathway inhibition in specific tumor types.

#### 6.3.2. XBP1 Peptide Vaccine: Inducing Specific Antitumor Immunity

Given the high expression of XBP1 in various tumors versus its low expression in normal tissues, XBP1-derived antigenic peptides have become ideal targets for cancer vaccine development. The core rationale of this strategy is to leverage the immunogenicity of XBP1 to actively elicit XBP1-specific cytotoxic T lymphocytes (CTLs) in vivo, thereby precisely eliminating tumor cells that highly express XBP1. Heteroclitic XBP1 peptides have been successfully identified for the two most common human leukocyte antigen (HLA) types, HLA-A2 and HLA-A24. These optimized heteroclitic peptides bind more effectively to major histocompatibility complex (MHC) molecules and induce potent XBP1-specific CTL responses. Studies have shown that these CTLs exhibit strong cytotoxic activity against various cancer cells, including multiple myeloma, breast cancer, colon cancer, and pancreatic cancer, demonstrating broad-spectrum antitumor potential [112,113]. Combining immunomodulators represents an effective strategy to further enhance vaccine efficacy. Lenalidomide, an immunomodulatory agent with intrinsic antitumor activity, has been shown to significantly augment the antitumor function of CTLs induced by the XBP1 peptide vaccine. Specifically, lenalidomide increases the proportion of central memory T cells, upregulates T cell activation markers and costimulatory molecules (such as CD28 and CD40L), and downregulates immune checkpoint molecules (such as CTLA-4 and PD-1), thereby promoting the proliferation, survival, and effector function of XBP1-specific CTLs for more effective tumor clearance [114]. These findings lay a solid foundation for the clinical application of XBP1 vaccines in combination with immunomodulators and illuminate a new direction for developing combination immunotherapies targeting XBP1-high tumors.

### 6.4. Challenges

Despite the promising therapeutic potential of targeting XBP1 in preclinical studies, the translation of these strategies into clinical practice faces a series of formidable challenges. The core of these challenges lies in the functional complexity of XBP1 under physiological and pathological conditions, its cell-type-specific effects, and the remarkable plasticity of tumor cells when confronted with stress. Therefore, a thorough understanding of the nature of these challenges is a prerequisite for designing effective and safe therapeutic regimens.

#### 6.4.1. Interference with Normal Physiological Functions and Potential Toxicity

As a core transcription factor of the UPR, XBP1 is not only critical for the survival of cancer cells but is also indispensable for the differentiation and functional maintenance of various normal cell types. For instance, XBP1 is essential for efficient antibody secretion by plasma cells [115] and for the differentiation of eosinophils [116] and intestinal Paneth cells [92]. Furthermore, XBP1 plays important roles in maintaining intestinal homeostasis, hepatocyte function, and the integrity of the hematopoietic stem cell pool [42,92]. Consequently, systemic inhibition of the XBP1 signaling pathway may disrupt these normal physiological processes, leading to unacceptable toxicities, such as immune deficiency, intestinal inflammation, or liver dysfunction. Indeed, studies have shown that in an acute liver injury model, hepatocyte-specific knockout of XBP1 unexpectedly exacerbated hepatocyte pyroptosis and liver damage, suggesting that XBP1 may exert tissue-protective effects under specific physiological or pathological contexts [3]. Thus, developing cell-type-specific (e.g., tumor-cell-targeted) XBP1 inhibitors or achieving precision intervention through local administration represents a critical strategy to overcome this challenge.

#### 6.4.2. Signaling Pathway Complexity and Cell-Type Specificity

The role of XBP1 can be diametrically opposite depending on the cell type or even the microenvironment of the same cell, posing a substantial challenge for therapy. In most cancer cells, XBP1 acts as a pro-survival and pro-proliferative factor [8,57,117]; however, in intestinal stem cells, XBP1 functions as a tumor suppressor, where its loss leads to stem cell expansion and spontaneous intestinal tumorigenesis [92]. Similarly, in hematopoietic stem cells, the IRE1α-XBP1 signaling axis restricts leukemogenesis, and its deletion promotes the progression of acute myeloid leukemia [42]. This functional duality necessitates that therapeutic strategies precisely distinguish whether the target is tumor cells or non-tumor cells, and that we deeply understand the regulatory effects of XBP1 on the functions of distinct immune cells within the specific tumor microenvironment. For example, although XBP1 promotes the immunosuppressive function of tumor-associated macrophages [20], sustained XBP1 activation in tumor-infiltrating T cells inhibits their mitochondrial respiration and antitumor function [77]. Therefore, the ultimate outcome of a seemingly straightforward “inhibit XBP1” strategy depends critically on which cell type is targeted and at what stage, thereby greatly increasing therapeutic complexity.

#### 6.4.3. Selectivity and Off-Target Effects of Inhibitors

To date, several small-molecule inhibitors have been developed to target the IRE1α-XBP1 pathway, including STF-083010 and MKC-8866, which inhibit the kinase/ribonuclease activity of IRE1α. Although these tool compounds are widely used in research, their specificity and potential off-target effects have not been fully elucidated. Some inhibitors may simultaneously affect other UPR branches, such as the PERK and ATF6 pathways, or interfere with other critical intracellular signaling cascades, thereby eliciting unintended biological effects. For instance, the classical chemotherapeutic agent doxorubicin was found to inhibit XBP1 activation, but subsequent studies revealed that its primary mechanism involves topoisomerase II inhibition rather than direct targeting of the IRE1α-XBP1 axis [103]. These findings serve as a cautionary note: before advancing inhibitors into clinical practice, their target specificity must be rigorously evaluated using advanced chemical and genetic tools to avoid erroneous conclusions and potential risks arising from non-specific effects.

#### 6.4.4. Compensatory Mechanisms and Adaptive Resistance

When subjected to single-agent targeted therapy, cancer cells frequently evade cell death by activating compensatory signaling pathways. Upon inhibition of the IRE1α-XBP1 pathway, tumor cells may upregulate other UPR branches, such as the PERK-ATF4 pathway [117], or activate alternative pro-survival signaling cascades, including NF-κB [118], to maintain cellular homeostasis and consequently develop drug resistance. For example, in colon cancer cells, even when XBP1 is inhibited, cells may sustain survival through the PERK-eIF2α pathway [117]. Furthermore, XBP1 itself is subject to complex feedback regulation; its expression is regulated by MYC, and in turn, XBP1 can form a transcriptional complex with MYC to jointly drive tumor growth [117]. These observations indicate that blocking the IRE1α-XBP1 pathway with a single agent may be insufficient for complete tumor eradication. Combining inhibitors targeting multiple UPR branches or integrating them with conventional chemotherapy or immunotherapy represents a promising strategy to overcome adaptive resistance and enhance therapeutic efficacy. Indeed, preclinical studies have demonstrated that combining IRE1α inhibitors with PARP inhibitors [110] or chemotherapeutic agents [58] produces synergistic anti-tumor effects. Therefore, exploring XBP1-based combination therapeutic strategies will be a critical direction for overcoming drug resistance and improving clinical outcomes.

In summary, although targeting the XBP1 pathway holds considerable therapeutic promise, its clinical translation remains fraught with challenges. Future research should focus on developing more selective inhibitors, exploring combination regimens to bypass compensatory mechanisms, and leveraging nanotechnologies or gene-editing tools to achieve cell-type-specific targeting. Only through multidisciplinary integration and refined intervention strategies can the theoretical advantages of targeting XBP1 be translated into tangible clinical benefits.

## 7. Conclusions and Perspectives

XBP1, a core transcription factor in the endoplasmic reticulum stress response, plays a complex and dualistic role in cancer development and progression. Accumulating evidence indicates that XBP1 not only serves as an intrinsic survival engine in tumor cells—driving proliferation, invasion, and metastasis through regulation of HIF1α, glycolysis, cell cycle progression, and apoptosis resistance—but also acts as a key modulator of immune cell functional reprogramming within the tumor microenvironment. This multi-level, multi-cell-type regulatory role positions XBP1 and its upstream IRE1α signaling axis as highly attractive “druggable” targets. However, most currently available pharmacological approaches act upstream through inhibition of IRE1α rather than through direct targeting of XBP1 itself. Translating XBP1-targeting approaches from bench to bedside faces several key challenges. For example, XBP1 exhibits tumor-suppressive functions in specific cell types, such as intestinal epithelial cells [92], highlighting its highly cell-type-specific and context-dependent activities. Furthermore, the two major isoforms of XBP1 display significant functional differences. XBP1-u not only serves as a precursor for XBP1-s but also independently participates in cholesterol metabolism and metabolic reprogramming by stabilizing SREBP2 or regulating mitochondrial localization [44,55], thereby adding complexity to targeted therapies. Accordingly, future research should focus on the following directions to translate XBP1-targeting strategies into effective clinical therapies.

### 7.1. Unraveling the Cell-Specific and Spatiotemporal Dynamics of XBP1 Function

Current research has revealed that XBP1 can exert context-dependent, and even opposing, functions in distinct cellular subsets. For instance, while XBP1 typically promotes survival in tumor cells, it can induce functional exhaustion in certain immune cells [77]. Future investigations must transcend conventional bulk analyses by employing single-cell sequencing, spatial transcriptomics, and conditional gene-knockout mouse models. These approaches will enable precise mapping of the functional landscape of XBP1 across different tumor types, distinct cellular subpopulations, and, importantly, at various stages of tumor progression. Elucidating these complex functional networks will lay a solid foundation for developing strategies that precisely modulate XBP1 activity in specific cell types, thereby circumventing the potential off-target toxicities associated with broad-spectrum inhibition. Only by clarifying these intricate functional networks can we achieve truly precision-targeted XBP1-based therapies.

### 7.2. Development of Highly Selective and Novel XBP1-Targeted Intervention Strategies

Current XBP1-targeting strategies primarily focus on inhibiting the endoribonuclease activity of its upstream regulator, IRE1α, using small-molecule inhibitors such as MKC8866 and STF-083010 [18,62]. However, the selectivity and potential off-target effects of these inhibitors require further optimization; consequently, developing more selective IRE1α inhibitors through structure-based drug design and high-throughput screening is an urgent priority. Furthermore, exploring strategies that directly target the transcriptional activity of XBP1s may offer more direct inhibitory effects. The development of novel intervention strategies, such as targeted degradation of xbp1, will provide additional avenues to overcome the specificity challenges inherent in XBP1-targeted therapy.

### 7.3. Exploration of XBP1-Based Combination Therapeutic Strategies

Given the extensive crosstalk between XBP1 and multiple oncogenic pathways, DNA damage repair pathways, and immune checkpoint pathways, XBP1 holds substantial potential as a target for combination therapy. Therefore, future research should focus on three key areas: first, mechanistic studies to elucidate how XBP1 inhibition remodels the tumor microenvironment, particularly the infiltration and function of immune cells, thereby providing a theoretical basis for combination with immunotherapy; second, optimization of combination regimens through systematic preclinical investigations to determine the optimal sequencing, dosage, and scheduling of XBP1 inhibitors with various therapies, maximizing synergistic efficacy while minimizing toxicity; and third, exploration of novel combination strategies, such as pairing XBP1 inhibitors with emerging therapies including metabolic interventions or epigenetic drugs, to overcome tumor resistance. Through multidimensional exploration of combination therapies, the full therapeutic potential of XBP1 targeting can be realized.

### 7.4. Developing Biomarkers to Accurately Screen Beneficiary Populations

The key to achieving precision therapy lies in the ability to predict which patients will benefit from XBP1-targeted treatment. Therefore, there is an urgent need to identify and validate predictive biomarkers of therapeutic response. Potential candidates include molecular markers, genetic background features, and immune microenvironment characteristics. Specifically, molecular markers may manifest as the expression level of XBP1s in tumor tissues or peripheral blood, the XBP1-u/XBP1-s ratio, or the expression signatures of its downstream target genes. Regarding genetic background, ARID1A mutation status may predict sensitivity to IRE1α inhibitors, whereas tumors with high MYC expression might exhibit a “synthetic lethal” dependency on XBP1 signaling. Furthermore, immune microenvironment features—such as specific immune cell infiltration patterns (e.g., the abundance of myeloid-derived suppressor cells or tumor-associated macrophage subsets) or the functional status of T cells—can also serve as predictive biomarkers. The establishment and validation of these biomarkers will provide essential tools for the “population stratification” required for XBP1-targeted therapy.

In summary, XBP1 serves as a critical hub linking cellular stress responses to malignant tumor progression. Research in this field has evolved from solely exploring molecular mechanisms to systematically dissecting the regulatory networks of the tumor microenvironment. Although challenges remain in achieving specificity, safety, and predictive efficacy in targeting XBP1, a deeper understanding of its complex roles in tumor development offers a compelling rationale that targeting XBP1 holds great promise as a novel direction in cancer therapy, ultimately bringing new hope to patients. Therefore, continued advancement of systematic research from basic science to clinical translation is essential to unlock the full therapeutic potential of XBP1.

## Figures and Tables

**Table 1:** Comparison of XBP1u and XBP1s isoforms.

Feature	XBP1u (Unspliced)	XBP1s (Spliced)
mRNA structure	Full-length mRNA containing a 26-nucleotide intron	Intron excised by IRE1α RNase, resulting in a frameshift
Protein length	precursor form	Spliced form
*C*-terminal sequence	Native *C*-terminal amino acid sequence	Distinct *C*-terminal sequence due to frameshift
Transcriptional activity	Minimal or weak transcriptional activity	Potent transcription factor with significantly enhanced transcriptional activation capacity
Primary function	Precursor to XBP1s; possesses unique biological functions independent of XBP1s, participating in autophagy and tumorigenesis	Activates downstream UPR target genes involved in protein folding, ERAD, ER biogenesis, and lipid metabolism; promotes cell survival and metabolic reprogramming
Role in cancer	Previously underestimated; now recognized as having independent roles in autophagy and tumorigenesis	Drives tumor cell survival, proliferation, metastasis, and therapy resistance through transcriptional regulation of multiple UPR target genes
Molecular switch	Serves as the inactive precursor awaiting splicing signal	Represents the “active” form that executes the adaptive transcriptional response to ER stress

**Table 2:** XBP1-mediated therapy resistance in different cancers.

Cancer Type	Therapy	XBP1-Mediated Mechanism	Therapeutic Implication
Non-small cell lung cancer	Cisplatin	CPSF6-mediated 3′UTR shortening of *XBP1* mRNA increases transcript stability, attenuating cisplatin-induced ER stress and promoting resistance.	Targeting CPSF6 or the 3′UTR regulation may restore chemosensitivity.
Colorectal cancer	Oxaliplatin	CircPDIA3/miR-449a/XBP1 positive feedback loop inhibits GSDME palmitoylation, blocking pyroptosis and driving resistance.	Interfering with this feedback loop could overcome oxaliplatin resistance.
Ovarian cancer	Cisplatin	Activation of IRE1α–XBP1 pathway; pharmacological inhibition of IRE1α reverses resistance.	IRE1α inhibitors may be combined with cisplatin to enhance efficacy.
Hepatocellular carcinoma	Sorafenib	XBP1 upregulates IL-6, activates STAT3, promoting proliferation and resistance to targeted therapy.	Combined blockade of XBP1 and IL-6/STAT3 may improve outcomes.
ER^+^ breast cancer	Tamoxifen	XBP1–ER positive feedback loop upregulates RRM2 and CDC6, driving endocrine resistance.	Targeting XBP1 or its effectors (RRM2/CDC6) could restore tamoxifen sensitivity.
HPV-negative oropharyngeal carcinoma	Radiotherapy	XBP1-induced IL-6 secretion activates STAT3, enhancing DNA double-strand break repair and conferring radioresistance.	Combining XBP1 inhibitors with radiotherapy may sensitize tumors.
Oropharyngeal carcinoma (EGFR-active)	Radiotherapy	EGFR potentiates IRE1α–XBP1–GRP78 and PERK–eIF2α–GRP94 arms, facilitating DNA repair and autophagy-mediated resistance.	Dual targeting of EGFR and IRE1α/XBP1 may overcome radioresistance.

## Data Availability

No new data were created or analyzed in this study.
